# Correlated compositional and mineralogical investigations at the Chang′e-3 landing site

**DOI:** 10.1038/ncomms9880

**Published:** 2015-12-22

**Authors:** Zongcheng Ling, Bradley L. Jolliff, Alian Wang, Chunlai Li, Jianzhong Liu, Jiang Zhang, Bo Li, Lingzhi Sun, Jian Chen, Long Xiao, Jianjun Liu, Xin Ren, Wenxi Peng, Huanyu Wang, Xingzhu Cui, Zhiping He, Jianyu Wang

**Affiliations:** 1 Shandong Provincial Key Laboratory of Optical Astronomy and Solar-Terrestrial Environment, Institute of Space Sciences, Shandong University, Weihai 264209, China; 2 Department of Earth & Planetary Sciences and McDonnell Center for the Space Sciences, Washington University, St Louis, Missouri 63130, USA; 3 Key Laboratory of Lunar and Deep Space Exploration, National Astronomical Observatories, Chinese Academy of Sciences, Beijing 100012, China; 4 Institute of Geochemistry, Chinese Academy of Sciences, Guiyang 550002, China; 5 Planetary Science Institute, School of Earth Sciences, China University of Geosciences, Wuhan 430074, China; 6 Institute of High Energy Physics, Chinese Academy of Sciences, Beijing 100049, China; 7 Key Laboratory of Space Active Opto-Electronics Technology, Shanghai Institute of Technical Physics, Chinese Academy of Science, Shanghai 200083, China

## Abstract

The chemical compositions of relatively young mare lava flows have implications for the late volcanism on the Moon. Here we report the composition of soil along the rim of a 450-m diameter fresh crater at the Chang′e-3 (CE-3) landing site, investigated by the Yutu rover with *in situ* APXS (Active Particle-induced X-ray Spectrometer) and VNIS (Visible and Near-infrared Imaging Spectrometer) measurements. Results indicate that this region's composition differs from other mare sample-return sites and is a new type of mare basalt not previously sampled, but consistent with remote sensing. The CE-3 regolith derived from olivine-normative basaltic rocks with high FeO/(FeO+MgO). Deconvolution of the VNIS data indicates abundant high-Ca ferropyroxene (augite and pigeonite) plus Fe-rich olivine. We infer from the regolith composition that the basaltic source rocks formed during late-stage magma-ocean differentiation when dense ferropyroxene-ilmenite cumulates sank and mixed with deeper, relatively ferroan olivine and orthopyroxene in a hybridized mantle source.

## Abstract (web summary)

The chemical compositions of young lava flows on the Moon have implications for late volcanism. Here, the authors present mineral distribution data from the Chang′e-3 Yutu rover in the northern Imbrium mare region, reporting unique compositional characteristics of a previously unsampled basalt type.

## Graphical abstract

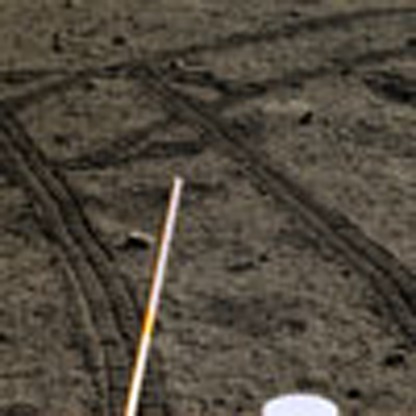

The combination of precise chemical and physical properties of Apollo and Luna samples with known locations provides ‘ground truth' for lunar remote sensing studies^1^,^2^. After some 40 years since the Apollo and Luna missions, China's Chang′e-3 (CE-3) landing and Yutu rover mission in December, 2013, provided the next robotic *in situ* measurements on the Moon^3^,^4^,^5^,^6^. The CE-3 landing site is in the northern part of the Imbrium basin (340.49°E, 44.12°N). Serial eruptions flooded the Imbrium basin from ∼3.5 Ga to ∼2 Ga (ref. 7), making this region of great scientific value for the detection of lava variations during extended volcanic activity^6^,^7^,^8^. The CE-3 landing site represents a relatively young (∼2.96 Ga) medium-Ti lunar basalt exposure^7^,^8^,^9^,^10^. The top Eratosthenian lava flows at the Mare Imbrium surface on which CE-3 landed are interpreted to be 10–60 m thick and up to 1,200 km in length^7^,^8^,^9^,^10^,^11^.

Here we combine the data from two payload elements of the Yutu rover, the Active Particle-induced X-ray Spectrometer (APXS) and Visible and Near-infrared Imaging Spectrometer (VNIS; see Methods section for the instrument descriptions), and report the composition and mineralogy of the region explored by the rover. Our analysis indicates that this young lunar mare region has unique compositional characteristics, and represents a new type of mare basalt that has not been sampled by previous Apollo and Luna missions and lunar meteorite collections. APXS data suggest that the regolith is extremely rich in FeO, high in CaO, intermediate in TiO_2_, modest in Al_2_O_3_ and poor in SiO_2_. We present the mineralogical information derived from APXS chemical data and VNIS spectral data, showing self-consistent and well-correlated results in mineral modes (for example, the proportions of high-Ca pyroxene, low-Ca pyroxene and olivine) and mineral chemistries (both data sets indicate an abundance of high-Ca ferropyroxene plus Fe-rich olivine). The more accurate *in situ* chemical and mineralogical measurements of the new basalt type provide ground-truth validation of remote sensing studies that also indicated the olivine-rich mineralogy of the basalt unit where CE-3 landed. Because the CE-3 landing site was on the ejecta of a fresh impact crater, we take the local regolith composition to be essentially that of the basalt excavated by the impact crater, and then consider the implications of the measured composition. Results suggest an origin from late-stage magma-ocean cumulates that crystallized after ilmenite saturation and hybridized with an olivine-rich cumulate. The CE-3 landing site and *in situ* analyses of the rocks and soils derived from the fresh crater near the landing site provide key new ground truth for some of the youngest volcanism on the Moon.

## Results

### Landing site description and Yutu rover operations

**Figure 1:**
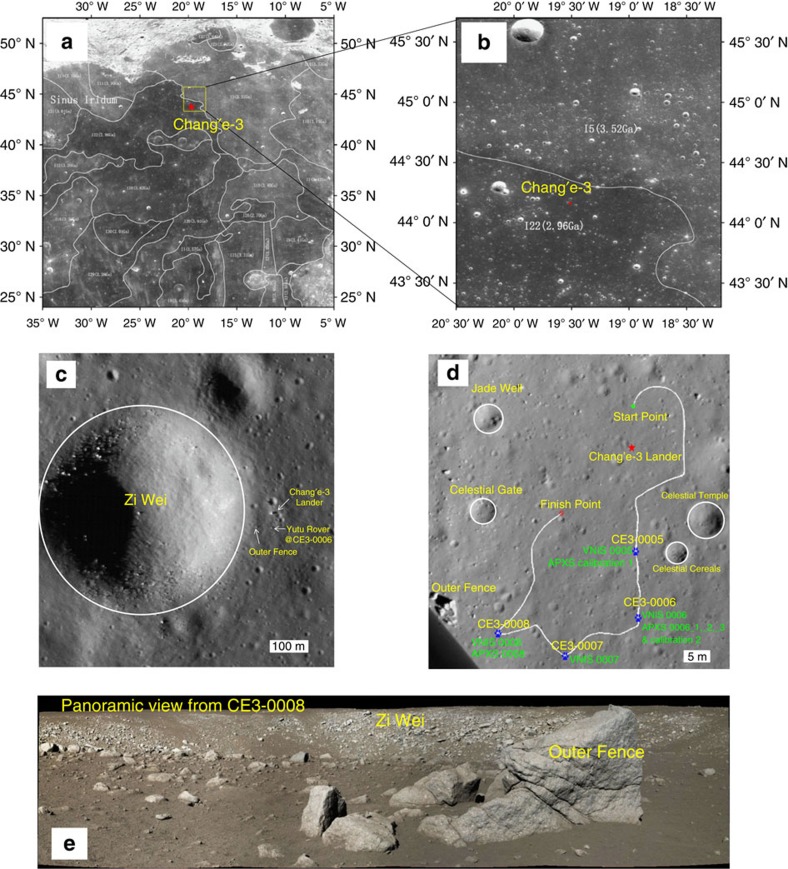
Location of the Chang'e-3 landing site. (**a**) Chang'e-1 CCD image with boundaries of typical mare basalt units^7^. (**b**) Chang'e-2 CCD image and (**c**) LROC NAC image (LROC NAC M1142582775R). (**d**) The traverse map of the Yutu rover and the locations of APXS and VNIS measurements. (**e**) Panoramic view of the ‘Zi Wei' crater by the Panoramic Camera on the Yutu rover at the CE3-0008 site.

The location of CE-3 from a global to a ground view is shown in Fig. 1. CE-3 landed on the rim of a young crater (∼27–80 Myr old^6^), initially informally named Purple Palace^4^ and now formally named Zi Wei (Fig. 1c). The diameter of the crater is ∼450 m, which would have excavated ∼40–50 m beneath the surface. The ejecta of the impact should cover the entire CE-3 landing site and the region explored by the Yutu rover, evidenced by the blocky surface seen by the landing camera (Fig. 1d) and big boulders encountered by Yutu during its traverse (Fig. 1d,e). The Panoramic Camera imaged two types of rocks; one is a mainly light-toned and coarse-grained rock and the other is a relatively darker, fine-grained rock (Supplementary Figs 1 and 2, Supplementary Note 1). During 32 days of surface operations, Yutu travelled 114 m in this region and made four sets of *in situ* and stand-off measurements at four locations (Fig. 1d)^3^,^4^,^6^. The APXS and VNIS aboard the Yutu rover acquired compositional and spectral measurements at four locations (CE3-0005, -0006, -0007 and -0008), as shown in Fig. 1d. A detailed description of the instruments, measurements and data processing are given in the Methods section and refs 12, 13, 14, 15, 16.

### Chemical compositions and normative mineralogies from APXS

**Figure 2:**
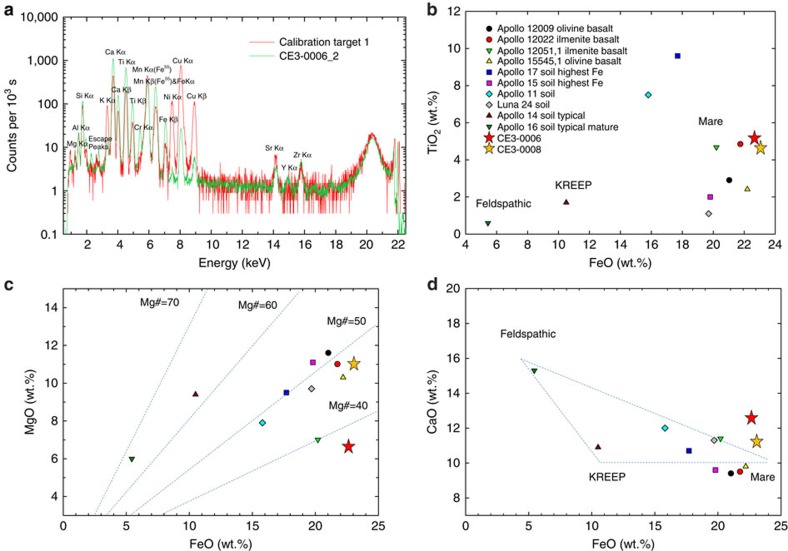
X-ray spectrum and chemical compositions of Chang'e-3 soils from APXS. (**a**) APXS spectrum CE3-0006_2 overlain on the calibration spectrum. Comparison of Chang'e-3 site surface soil samples with Apollo and Luna samples^2^,^17^ in (**b**) FeO versus TiO_2_, (**c**) FeO versus MgO and (**d**) FeO versus CaO.

APXS spectra show peaks of Mg, Al, Si, K, Ca, Ti, Cr, Fe, Ni, Sr, Zr and Y from the CE-3 soils (Fig. 2a). We used the peak-area ratio of measured samples and the calibration target (Supplementary Table 1) to derive the chemical compositions of three measured soils (CE3-0006_2, -0006_3 and -0008, Table 1). In general, the major element concentrations of the three soils at the CE-3 landing site are similar to each other and represent a distinctive composition (Table 1, Fig. 2b–d). They are characterized by low SiO_2_ (∼41.2 wt.%), very high FeO (∼22.8 wt.%), high CaO (∼12.1 wt.%), intermediate TiO_2_ (∼5.0 wt.%) and modest Al_2_O_3_ (∼9.7 wt.%).

When compared with Apollo and Luna soils and basaltic rocks (Fig. 2b–d)^2^,^17^, the TiO_2_ versus FeO relation of the CE-3 soils bears some similarity with Apollo 12 ilmenite basalts, but CE-3 soils have higher FeO and TiO_2_ (Fig. 2b). The CE-3 soils have MgO concentrations in the range 6.3–11.0 wt.% (Table 1) with derived Mg# (=Mg/(Mg+Fe) × 100)<50 (Fig. 2c) at the low end, but a higher CaO compared with other mare samples (Fig. 2d), and deviating from the KREEP—feldspathic highlands—mare compositional triangle based on the returned lunar samples. These compositional features suggest that the CE-3 soils differ significantly from other known lunar basaltic materials.

On the basis of chemical composition (Table 1), we calculated the abundances of normative minerals of CE-3 soils using a CIPW (Cross, Iddings, Pirsson and Washington) norm. The major CIPW norm results are summarized in Table 1 and the detailed results are shown in Supplementary Tables 2,3. For CE3-0006_2 and CE3-0006_3 (sampling sites ∼10 cm apart), we calculated the mean value as ‘Mean_0006.' For the norms summarized in Table 1, we combined the high-Ca pyroxene components as diopside (Di) and the Ca-poor components as hypersthene (Hy). Given the analytical uncertainties associated with the APXS data (Supplementary Table 4), the main difference is in MgO, which is significantly higher in CE3-0008 (11 wt.%) compared with CE3-0006 (6.7 wt.%). The difference in MgO translates to a difference in the relative abundance of olivine and pyroxene and in the ratio of Di to Hy (Table 1). The higher MgO concentration of the CE3-0008 soil results in a higher Mg# (46) compared with CE3-0006 (34) and a higher Fo (=Mg/(Mg+Fe) × 100) in olivine, that is, ∼Fo_51_ for the CE3-0008 soil and ∼Fo_40_ for the CE3-0006 soil. The CE3-0006 soil is also richer in the high-Fe endmember for both Di and Hy (Table 1) as a result of the difference in MgO. Considering analytical uncertainties for Al, Si, Ca, Fe and Ti, normative abundances of plagioclase and ilmenite are the same in -0006 and -0008, within analytical uncertainties.

In Table 1, the ‘Means_all' column shows the average chemical composition and normative mineralogy summary of the CE-3 landing site soils. The soils have a high percentage of normative pyroxene (∼42 wt.%), with most being high-Ca pyroxene, Di (29 wt.%), that is, about two times the Hy (13 wt.%). The normative feldspar content (27 wt.%) is within the range of many lunar basaltic samples. The normative olivine content (20 wt.%, corresponding to 17 vol.%) of CE-3 is at the high end of the range for known lunar basalts (for example, Apollo 12 olivine basalt has ∼20 vol.% olivine^2^). In the CE-3 soils, olivine is Fe-rich with relatively low average Fo content (∼43). The normative ilmenite contents of the three CE-3 soils are similar, averaging ∼9 wt.%. The average Mg# of the soils is ∼38, indicating the exceptionally ferroan character of source rocks that make up the local surface soils.

### Mineral chemistry and mineral modes based on VNIR spectra

**Figure 3:**
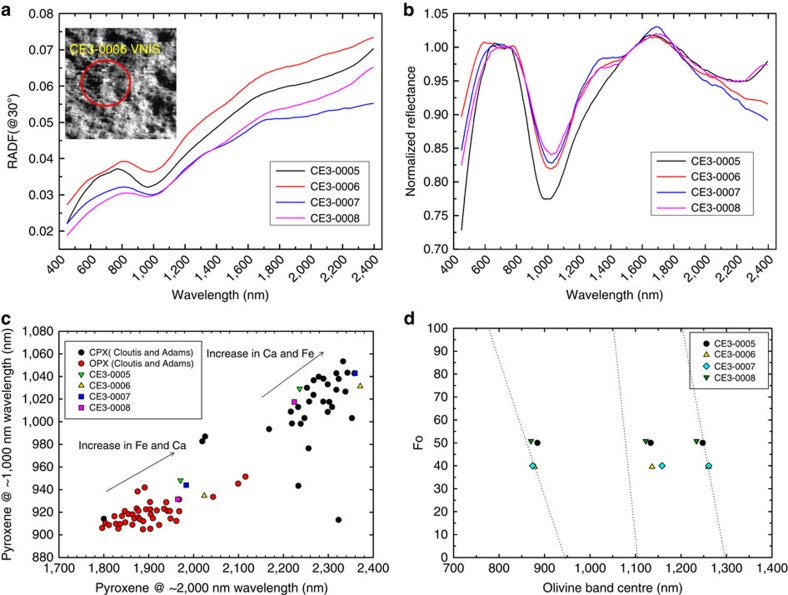
Visible-NIR spectral properties and mineral chemistry of Chang'e-3 soils from VNIS. (**a**) Combined VNIS spectra (450–2,400 nm) from sites 0005, 0006, 0007 and 0008. The inset image is from site CE3-0006 of the VNIS (450–950 nm) image mode at 750 nm. The dashed circle indicates the region measured by the VNIS-point spectral mode (900–2,400 nm). (**b**) VNIS spectra after continuum removal. (**c**) Pyroxene VNIS peak positions of the CE-3 soils overlain on experimental results from Adams^27^ and Cloutis and Gaffey^28^. (**d**) Fo values of olivine in four CE-3 soils derived from VNIS spectra, overlain on calibration lines (Sunshine and Pieters^20^).

The visible-NIR (near-infrared) spectra (Fig. 3a,b) of four VNIS observations show characteristic 1 and 2 μm absorption features owing to the electron transfer of Fe^2+^ in the M1 and M2 sites of lunar mafic silicates^18^. The spectra (Fig. 3a) have obvious absorption features and relatively flat profiles, indicating a low degree of space weathering, consistent with the fact that the CE-3 landing site sits on a relatively young Eratosthenian lava flow and the fresh ejecta of the young and fresh Zi Wei crater.

To estimate the average composition of minerals contributing to the spectra, we apply the modified Gaussian model (MGM)^19^,^20^ to deconvolve the spectral bands. We find that the spectra from CE3-0005 and CE3-0008 sites have wide and strong 1 μm absorption bands but shallow 2 μm band depths (Fig. 3a,b), thus they should have a higher 1–2μm band area ratio (BAR), which implies the presence of a significant amount of olivine in the soils of these two sites^15^,^21^,^22^,^23^. The absorption components of all four continuum-removed spectra (Fig. 3b) are calculated using MGM, as mixtures of three endmembers, high-Ca pyroxene (HCP), low-Ca pyroxene (LCP) and olivine (Supplementary Note 5, Table 2). This combination is the most complicated for this type of spectral deconvolution^24^,^25^,^26^. The results of the MGM deconvolution are shown in Table 2.

Extensive laboratory studies of terrestrial and synthetic pyroxenes provide the basis to correlate the 1- and 2-μm band positions with their chemical compositions^27^,^28^,^29^,^30^. We plot the central positions of deconvolved 1 and 2 μm bands from HCP and LCP components based on the data of Adams^27^ and Cloutis and Gaffey^28^ (Fig. 3c). Here we define the HCP as wollastonite (Wo)>30 and LCP as Wo<30, keeping with previous work by Sunshine *et al*.^31^ and Klima *et al*.^30^. By comparison, the compositional features of LCP of the four soils are similar (Fig. 3c) and very Fe-rich, relative to orthopyroxene examined by Adams^27^ and Cloutis and Gaffey^28^. However, the HCP compositions of CE-3 soils occur in two groups; CE3-0006 and CE3-0007 are slightly richer in Ca and in Fe than CE3-0005 and CE3-0008, consistent with APXS data (Table 1). The pyroxene chemistry of the CE-3 soils derived from VNIS data thus supports their general Fe-rich character, with CE3-0006 and CE3-0007 having even higher Fe contents, consistent with APXS results.

The volume percentage ratio of HCP and LCP (HCP/LCP) can be estimated using the band-strength ratios of 1 and 2 μm bands from MGM deconvolution of VNIS spectra^19^,^24^,^29^,^30^,^32^. The HCP/LCP vol.% ratios for four CE-3 soils were calculated using both the 1- and 2-μm band-strength ratios. The results for each soil using two ratios are consistent (Table 2), indicating an equivalent compositional effect on both 1 and 2 μm bands. Overall, the HCP/LCP ratios in four CE-3 soils are similar, with HCP about two times LCP in abundance.

The MGM-derived band positions of olivine shift as a function of Fo content, thus they can be used to estimate olivine chemistry^20^,^25^. We plot central positions of two deconvolved M1 component bands of CE-3 olivine (Fig. 3d) with trend lines determined on terrestrial samples by Sunshine and Pieters^20^. The central positions of the two olivine M1 bands occur at 870–884 nm and 1234–1261, nm for the four CE-3 soils, suggesting they are Fe-rich (30<Fo<55, Fig. 3d). Specifically, spectra indicate that CE3-0005 and CE3-0008 soils have higher olivine Fo values than the other two soils.

The precise location of the M2 band (∼1050, nm) of olivine in the VNIS spectrum is difficult to determine via MGM deconvolution^20^,^33^ (Supplementary Note 5). However, we plotted the central positions of olivine M2 bands of four CE-3 soils derived from MGM deconvolution in Fig. 3d, which also shows a trend along the trend line determined by Sunshine and Pieters^20^. Therefore, olivine chemistry of the CE-3 soils derived from VNIS data supports their general Fe-rich character, consistent with normative analysis of the APXS results (Fo ∼43 on average, Table 1). The band-strength ratios of the HCP 1-μm band to the olivine M1 band near 1.25 μm could also be used to estimate their volume percentage^24^. The four CE-3 soils can be divided into two groups (Table 2): the CE3-0005 and -0008 soils are richer in olivine on the basis of VNIS analysis (HCP/OL=2.0 and 2.3) than CE3-0006 and CE3-0007 (HCP/OL=3.0 and 3.3, respectively).

### Refinement of mineral mode by correlated APXS and VNIS

A key result from both the APXS and VNIS data is the inferred abundance of olivine. The APXS data indicate relatively low SiO_2_ and high FeO+MgO, resulting in a significant proportion of olivine in the norm (10 vol.% in CE3-0006 and 30 vol.% in CE3-0008, Table 1). Our MGM analysis of the VNIS spectra also reflects high olivine contents (the HCP/OL ratio is 3.0 for CE3-0006 and 2.3 for CE3-0008, Table 2). A high olivine content coupled with intermediate to high TiO_2_ makes the CE-3 soil and the basalt from which it derives unique among the known lunar samples, similar to a basalt type that has been inferred from orbital data^34^, but not until the CE-3 mission verified by *in situ* or sample analysis.

Second, olivine chemistry derived from the norm analysis based on the APXS composition (Table 1) and from the central positions of the olivine M1 bands in VNIS spectra (Fig. 3d) both support the Fe-rich character of olivine in CE-3 soils. Fe-rich olivine was predicted on the basis of remote sensing of this area^21^, thus the Fe-rich olivine found by CE-3 indicates a relatively evolved magma from late-stage volcanic activity in the Imbrium basin^35^.

We also find similarity in pyroxene features inferred from normative analyses of the APXS composition (Table 1) and deconvolved VNIS spectra (Table 2). For example, both sets of analyses suggest CE-3 soils are rich in HCP. A good match was found between HCP/LCP ratios in CE3-0006 soil derived from VNIS (2.0, Table 2) and the Di/Hy components derived from APXS analysis (2.0, Table 1). The CIPW norm analysis based on APXS composition indicates a much higher Di/Hy ratio for CE3-0008 (8.7, Table 1) than the HCP/LCP derived from VNIS (2.3, Table 2). This large difference results in part from the effect of the normative pyroxene components in which the Hy component has no Ca. In reality the LCP pyroxene (in this case, pigeonite) does contain Ca. The effect is greatest in CE3-0008 because it contains so much olivine that there is little normative Hy and abundant Di. Moreover, the VNIS and APXS target areas were within a short distance of each other (<1 m), so we do not expect such a large variation in this less (space-) weathered basaltic regolith.

To provide a better comparison between APXS data and VNIS data, we use typical compositions of lunar mare minerals (that is, olivine, augite, pigeonite and plagioclase) as endmembers in a mixing-model calculation instead of the normative calculation results (Supplementary Table 7). The results of this mixing analysis are shown in Table 3.

From the mixing analysis, the refined mineral mode of CE3-0008 yields an Aug/Pig ratio of 2.4 (Table 3), which matches well with the VNIS HCP/LCP ratio (2.3, Table 2) based on VNIS spectral deconvolution. For CE3-0006, the Aug/Pig value in the refined mineral mode (2.0) and the HCP/LCP value in the VNIS-derived mode (2.0) are essentially the same. Considering these results, the APXS and VNIS mineral modal data are consistent.

The CE3-0008 soil may have a greater abundance of material contributed from deeper levels of the nearby Zi Wei crater (Fig. 1e), with a composition similar to the nearby light-toned rocks (Supplementary Figs 2 and 3) such as the ‘Outer Fence' boulder. From an image-based mineral modal estimation of Outer Fence, we infer ∼20 vol.% in plagioclase phenocrysts (Supplementary Fig. 3, Supplementary Table 5, Supplementary Note 2), which is in general agreement with our estimations (∼33 vol.%) of nearby CE3-0008 soil. Moreover, the regolith reflectance differences observed by the Lunar Reconnaissance Orbiter Camera Narrow Angle Cameras (LROC NAC) are rather limited around the landing site (Supplementary Fig. 4, Supplementary Note 3). The Al_2_O_3_ content of CE-3 landing site based on experience of lunar samples is most likely in the range of 7–10 wt.% (Supplementary Fig. 5, Supplementary Note 4). The reflectance of the nearby rocks at the CE-3 landing site may result from texture-related human-eye brightness exaggeration (that is, some workers^36^ initially interpreted the rock as an aluminous basalt with a plagioclase content possibly exceeding 40 vol.% (see Supplementary Notes 2–4 for additional discussion).

### Correspondence between the landing site and remote sensing

Lunar Prospector (LP) Gamma-Ray Spectrometer (GRS) results suggest that soils developed on north-central Imbrium mare basalts, including the CE-3 landing site, have high FeO (>20 wt.%, half-degree per pixel binning) and TiO_2_ (5.20 wt.%, 2° per pixel binning)^6^,^37^. Clementine data for areas near the CE-3 site indicate ∼19 wt.% FeO and 5–7 wt.% TiO_2_. The LP-GRS TiO_2_ data in this part of Imbrium are variable, however. Considering both the LP-GRS data and the Clementine ultraviolet–visible data, the values are broadly similar to those of some widespread western Procellarum mare regions (for example, centred ∼18° N and 303° E) where the surface is spectrally similar and where LP-GRS TiO_2_ values are similar (4–6 wt.%). TiO_2_ is normally used as the primary compositional criterion to classify remote sensing data for lunar basalts and indicates that many of the lunar basalts are actually intermediate in TiO_2_ content (for example, 4.5–7.5 wt.%)^38^. The CE-3 composition falls in this range, thus we regard them as intermediate Ti basalt (similar to Neal *et al*.^36^). The intermediate Ti content of CE-3 provides an important calibration point for the TiO_2_ estimation by spectral data (for example, Clementine UVVIS)^38^,^39^, which has been problematic (for example, Gillis *et al*.^40^ suggested about 50% uncertainty in the Imbrium basalts). The CE-3 TiO_2_ data provide ground truth for further revision or evaluation of TiO_2_ estimation methods for remote sensing studies. The high CaO content of the CE-3 soils (compared with other mare basalts, Fig. 2d) and the abundance of HCP inferred from the visible-NIR spectra support a high Ca content in the area of this flow unit. This result is consistent with the LP-GRS results (CaO of ∼11.1 wt.%, same region in north-central Imbrium at 2° per pixel^37^) and Clementine UVVIS mineral mapping (rich in clinopyroxene and olivine, but poor in orthopyroxene)^2^,^41^. LRO Diviner data^42^ show a Christiansen feature value near 8.55 μm, indicating a low SiO_2_ content in this region, which qualitatively agrees with the low SiO_2_ (41.2 wt.% average, Table 1) of the CE-3 APXS measurements. Moreover, the high olivine content and Ti enrichment in the late-stage volcanism of the Procellarum and Imbrium regions have been interpreted by many authors^21^,^22^,^23^,^34^,^43^,^44^ according to spectral data from telescopic observations, the UVVIS camera on Clementine, and the Moon Mineralogy Mapper (M^3^) on Chandrayaan-1, and these observations are supported in the Imbrium region by CE-3 results.

## Discussion

Given a basalt that is rich in TiO_2_ (5%) yet also rich in olivine (>10%), we consider its possible origin. The composition of CE-3 basalt, inferred from APXS analyses of immature soils at the site derived from the uppermost young basalt flow, is FeO-rich, with an Mg/(Mg+Fe) value of ∼0.4. This composition suggests derivation from late-stage magma-ocean cumulates that crystallized after ilmenite saturation and thus were rich in ferropyroxene and ilmenite, possibly hybridized with an intermediate to late-stage olivine-orthopyroxene cumulate. Such an origin may be common among late-stage basalts in the Procellarum-Imbrium region (Procellarum KREEP Terrane or ‘PKT'^45^,^46^), producing olivine-bearing, ilmenite-rich ferrobasalts such as those of the flow sampled by CE-3 in north-central Imbrium as well as the extensive Ti-rich, olivine-bearing basalts of Western Procellarum^21^,^34^. Extended magmatic activity in the PKT may have been driven by radioactive decay and heating of late-stage, incompatible-element-rich cumulates that were also rich in Ti. Olivine enrichment would not be expected with such cumulates because lunar magma ocean (LMO) residual melts at the stage of ilmenite saturation (>95% LMO crystallization) would most likely be saturated with pigeonite and augite^47^,^48^, thus requiring localized mixing or hybridization with an olivine-rich cumulate. In that case, the olivine-rich cumulate would, itself, be fairly iron-rich, for example, as might have been produced from the LMO after some 75% solidification. These would be late-stage LMO cumulates, likely enriched in incompatible trace elements (ITEs), including U and Th, consistent with prolonged mare basaltic activity in this part of the Moon^49^. An origin of such hybridized magmas, as formed by partial melting of upper mantle cumulates, is similar to the origin inferred by Snyder *et al*.^47^ for high-Ti basalts.

In conclusion, from a correlated analysis of the regolith derived from rocks at the CE-3 landing site, freshly excavated by Zi Wei crater, we recognize a new type of lunar basalt with a distinctive mineral assemblage compared with the samples from Apollo and Luna, and the lunar meteorites. The chemical and mineralogical information of the CE-3 landing site provides new ground truth for some of the youngest volcanism on the Moon.

## Methods

### Instruments and data descriptions

The APXS is designed to conduct *in situ* elemental measurements of lunar regolith, using ^55^Fe and ^109^Cd as the excitation sources. The APXS is installed on the arm of the Yutu rover with an effective detection area of ∼50 mm in diameter^13^. The VNIS employs the Acousto-Optic Tunable Filter (AOTF) technique to provide hyperspectral images in the 450–950-nm region and point spectral measurements in the 900–2,400-nm region^14^,^15^,^16^. The VNIS is installed on the front of the rover at a height of 0.69 m, observing the lunar surface at a fixed 45° view angle. The targeting area of the VNIS imager is ∼16 × 21 cm. The targeting area of the VNIS-point spectrometer is a circle of ∼7 cm diameter, inside of the targeting area (Fig. 3a) of the VNIS imager. The distance between the sampling areas of the APXS and VNIS at one location is within 1 m. The detailed descriptions of these two instruments and their calibration procedures can be found in refs 12, 13, 14, 15, 16.

This study is based on level 2C (APXS) and 2B (VNIS) data released by the Ground and Research Application System (GRAS) of the Chang′e-3 Project. The APXS conducted two calibration target measurements and four sets of lunar soil measurements at two locations (CE3-0006 and -0008). Measurement 0006_1 was a test measurement at ∼5 cm distance from the surface of the regolith, 0006_2 was at the same location as 0006_1 but at a distance of ∼2.5 cm above the surface and 0006_3 was at a lateral distance of about 10 cm from 0006_2. The VNIS acquired four hyperspectral images (450–950 nm) and four point NIR spectral measurements (900–2,400 nm) at four locations (CE3-0005, -0006, -0007 and -0008). The locations of the APXS and VNIS measurements are shown in Fig. 1d. The APXS data at CE3-0006_1-3 and CE3-0008 correspond to the VNIS spectra from CE3-0006 and CE3-0008.

### APXS data processing and analysis

The APXS has a calibration target with known chemical composition (Fig. 2a). We employed the peak-area ratio of measured samples and calibration target (proportional to the elemental ratios of corresponding elements) to derive the chemical compositions of the CE-3 soils. Our APXS data processing began with level 2C data, which has undergone energy, dead-time and temperature corrections^13^. The four raw spectra were accumulated for 2657, s, 2778, s, 2050, s and 3627, s. We first normalized the spectral counts of all four APXS spectra to 10^3^ seconds (Fig. 2a). The background was then removed from raw spectra to eliminate the effects of multiple scattering. The peak areas of individual elements were derived by spectral curve fitting using GRAMS software (Galactic Industries Corporation, NH, USA). A Gaussian function was used to fit the peak shape. Multiple iterations in curve fitting were conducted until convergence was reached. The resulting peak area for each element was then divided by that from the calibration target, and multiplied by the known concentration of that element in the calibration target, to obtain the nominal concentrations of elements. The concentrations of seven major elements (Mg, Al, Si, K, Ca, Ti and Fe) as oxides were then normalized to 99% to obtain the final concentration of each of these elements in a CE-3 surface sample, with the remaining 1% set for elements not measured but known to be present, including Cr_2_O_3_, MnO, Na_2_O and P_2_O_5_, to 0.3, 0.3, 0.3 and 0.1 wt.%, respectively (Table 1). This method has the advantage of normalization of different observation geometries and instrument effects (for example, the CE3-0006_1 test measurement also shows a relatively reasonable result compared with CE3-0006_2, although it was taken at a distance of ∼2.5 cm higher than the other three measurements). Neal *et al*.^36^ also reported derived chemical compositions of the CE-3 soils, but using instead a Fundamental Parameters Method. Their results are in general agreement with our derived composition, but differ in detail, especially for Al. A detailed analysis of the differences is in the Discussion section of this paper.

### VNIS data processing and analysis

Our VNIS spectral data processing began with level 2B data, which has undergone dark current, flat-field, temperature corrections and radiometric and geometric calibration^15^. For each of the four locations, we averaged ∼9182 pixels spectra (450–950 nm) of the sampling area from VNIS in the image mode, then connected the averaged spectrum with a point spectrum (900–2400, nm) measured from a circular area within the imaged area (Fig. 3a). To correct the step artifacts between the two spectral ranges due to the response differences of the two detectors, a factor obtained from 900 nm of the point spectrum was used to multiply the averaged spectrum in the 450–900 nm region. We derived the single scattering albedo of the combined spectra, which is independent of the illumination geometry of the visible-NIR measurements. Based on the Hapke radiative transfer model^50^,^51^, the radiance factor was converted at the standard illumination geometry (*i*=30°, *e*=0° and *α*=30°) to facilitate comparison. The spectra were then smoothed using the Savitzky–Golay smoothing method^52^, which is a spectral smoothing algorithm that essentially performs a polynomial regression to the data points in a moving window (Fig. 3b). The spectral background (continuum) was removed using a straight line that connects the spectral points at 750 nm and 1700, nm. Mineral modes of four CE-3 soils were extracted by spectral deconvolution using the MGM (refs 19, 20, 24, 25, 26, 32; Supplementary Figs 6 and 7, Supplementary Table 6, Supplementary Note 5). The software was downloaded from the Reflectance Experiment Laboratory (http://www.planetary.brown.edu/mgm/).

## Additional information

**How to cite this article:** Ling, Z. *et al*. Correlated compositional and mineralogical investigations at the Chang′e-3 landing site. *Nat. Commun.* 6:8880 doi: 10.1038/ncomms9880 (2015).

## Supplementary Material

Supplementary Information Supplementary Figures 1-7, Supplementary Tables 1-8, Supplementary Notes 1-5 and Supplementary References.

## Figures and Tables

**Table 1:** Compositional data in weight percent and results of CIPW norm of Chang'e-3 soils from Yutu APXS after calibration.

	**CE3-0006_1**†	**CE3-0006_2**	**CE3-0006_3**	**CE3-0008**	**Mean_0006**†	**Mean_all**†
SiO_2_	41.8±3.1	42.3±3.2	41.6±3.1	39.6±3.0	42.0±3.1	41.2±3.1
TiO_2_	5.0±0.1	5.2±0.1	5.1±0.1	4.6±0.1	5.2±0.1	5.0±0.1
Al_2_O_3_	10.0±0.7	9.8±0.6	10.0±0.7	9.3±0.6	9.9±0.7	9.7±0.6
Cr_2_O_3_*	*0.3*	*0.3*	*0.3*	*0.3*	*0.3*	*0.3*
FeO	21.7±2.0	22.7±2.1	22.6±2.1	23.0±2.1	22.6±2.1	22.8±2.1
MnO*	*0.3*	*0.3*	*0.3*	*0.3*	*0.3*	*0.3*
MgO	8.1±0.1	6.3±0.1	7.0±0.1	11.0±0.1	6.7±0.1	8.1±0.1
CaO	12.3±0.4	12.6±0.4	12.6±0.4	11.2±0.3	12.6±0.4	12.1±0.4
Na_2_O*	*0.3*	*0.3*	*0.3*	*0.3*	*0.3*	*0.3*
K_2_O	0.11±0.01	0.12±0.01	0.11±0.01	0.11±0.01	0.12±0.01	0.11±0.01
P_2_O_5_*	*0.1*	*0.1*	*0.1*	*0.1*	*0.1*	*0.1*
						
Plag (wt.%)	28	27	28	26	28	27
Di (wt.%)‡	29	31	31	26	31	29
Hy (wt.%)‡	15	20	15	3	18	13
∑ Px (wt.%)	45	52	46	29	49	42
Ol (wt.%)	16	10	15	34	12	20
Ilm (wt.%)	10	10	10	9	10	9
Plag (vol.%)	34	33	34	32	33	33
Di (vol.%)	31	33	32	28	33	31
Hy (vol.%)	14	18	14	3	16	12
∑ Px (vol.%)	45	51	46	31	49	43
Ol (vol.%)	14	8	12	30	10	17
Ilm (vol.%)	7	7	7	6	7	7
Plag An	88	88	88	88	88	88
Oliv Fo	46	38	41	51	40	43
Mg#	40	33	36	46	34	38

Among the four locations, only two (CE3-0006 and CE3-0008) were analysed by APXS.

^*^The compositional data are normalized to 99% to allow for missing elements. (Missing elements Cr_2_O_3_, MnO, Na_2_O and P_2_O_5_ are set to 0.3, 0.3, 0.3 and 0.1 wt.% for more reasonable calculations of CIPW norms. Fe^3+^/Fe_Total_ are assumed to be 0.0 for lunar minerals for the CIPW calculations.

^†^CE3-0006_1 is for reference in the test mode with a detection distance of ∼5 cm above the surface soils, and thus is not included in the calculation of Mean_0006). Mean_0006 is the average value of CE3-0006_2 and CE3-0006_3. Mean_all is the average value of CE3-0006_2 and CE3-0006_3 and CE3-0008. Among the four locations, only two (CE3-0006 and CE3-0008) were analysed by APXS.

^‡^In the CIPW calculation, we refer to the sum of HCP components (Di Wo, Di En and Di Fs) as Di, whereas the Hy is the sum of Hy En and Hy Fs. Mg#, 100Mg/(Mg+Fe) atomic.

**Table 2:** MGM results of the four VNIS spectra.

**Spectrum**	**CE3-0005**			**CE3-0006**		
**Mineral**	**Centre**	**FWHM**	**Strength**	**Centre**	**FWHM**	**Strength**
Olivine	884	138	−0.103	878	135	−0.026
	1,134	139	−0.076	1,136	122	−0.073
	1,248	291	−0.099	1,260	237	−0.059
HCP‡	1,029	144	−0.196	1,031	145	−0.179
	2,236	337	−0.070	2,371	404	−0.099
LCP‡	948	110	−0.106	935	122	−0.091
	1,971	240	−0.035	2,024	373	−0.051
HCP/LCP* (1 μm ratio)	1.8 (LCP/(LCP+HCP)≈0.35)			2.0 (LCP/(LCP+HCP)≈0.34)		
HCP/LCP* (2 μm ratio)	2.0 (LCP/(LCP+HCP)≈0.33)			1.9 (LCP/(LCP+HCP)≈0.34)		
HCP/OL† (1 μm/1.25 μm)	2.0 (OL/(OL+HCP)≈0.34)			3.0 (OL/(OL+HCP)≈0.25)		
						
**Spectrum**	**CE3-0007**			**CE3-0008**		
**Mineral**	**Centre**	**FWHM**	**Strength**	**Centre**	**FWHM**	**Strength**
Olivine	874	148	−0.038	870	136	−0.037
	1,158	121	−0.045	1,123	146	−0.068
	1,261	251	−0.053	1,234	268	−0.063
HCP‡	1,043	166	−0.175	1,017	142	−0.144
	2,359	429	−0.145	2,224	395	−0.069
LCP‡	944	130	−0.076	932	111	−0.060
	1,983	309	−0.065	1,965	271	−0.030
HCP/LCP* (1 μm ratio)	2.3 (LCP/(LCP+HCP)≈0.30)			2.4 (LCP/(LCP+HCP)≈0.29)		
HCP/LCP* (2 μm ratio)	2.2(LCP/(LCP+HCP)≈0.31)			2.3 (LCP/(LCP+HCP)≈0.30)		
HCP/OL† (1 μm/1.25 μm)	3.3 (OL/(OL+HCP)≈0.23)			2.3 (OL/(OL+HCP)≈0.30)		

^*^The HCP/LCP is the volume ratio of HCP to LCP, determined by the 1- and 2-μm band-strength ratio for the two phases. The normalized band-strength ratios, LCP/(HCP+LCP), indicating the fractions of LCP in the mixtures, are also given for references in brackets. The consistencies in LCP/(LCP+HCP)s for the 1- and 2-μm bands are important in showing that olivine and other mineral absorptions are not skewing the MGM results^32^.

^†^The HCP/OL is the volume ratio of HCP to olivine, determined by the peak strength ratio for the two phases (1 μm peak of HCP versus ∼1.25 μm peak of olivine). The normalized band-strength ratios, OL/(HCP+OL), are also indicated in brackets.

^‡^Spectrally, HCP has the maximum absorption of the 1-μm feature located beyond 980 nm and the 2-μm feature, beyond 2,200 nm, whereas LCP has these features located at <980 nm and 2,200 nm, respectively.

**Table 3:** Mineralogy of Chang'e-3 soils derived from APXS data using mixture modelling of the chemical composition.

**APXS Mixing components**	**CE3-0006_1**	**CE3-0006_2**	**CE3-0006_3**	**CE3-0008**	**Mean_0006**‡	**Mean_all**‡
Olivine	11.9	9.2	10.5	23.4	9.8	14.4
Augite	36.4	37.1	36.2	31.6	36.6	35.0
Pigeonite	16.9	18.4	17.9	13.2	18.2	16.5
Plagioclase	27.7	27.4	27.8	25.8	27.6	27.0
Ilmenite	8.0	8.3	8.5	7.7	8.4	8.2
Apatite*	*0.2*	*0.2*	*0.2*	*0.2*	*0.2*	*0.2*
Cr-spinel*	*−0.1*	*0.1*	*0.0*	*-0.2*	*0.0*	*0.0*
Sum	101.2	100.7	101.1	101.8	100.9	101.2
Olivine_chem	Fo38.2	Fo34.7	Fo36.1	Fo49.6	Fo35.4	Fo40.1
Augite_chem	En30.1Wo33.7Fs36.2	En24.8Wo34.9Fs40.6	En30.5Wo33.7Fs35.8	En39.5Wo32.2Fs28.4	En27.7Wo34.3Fs38.2	En31.6Wo33.6Fs34.9
Pigeonite_chem	En35.7Wo20.1Fs44.2	En28.1Wo21.0Fs50.9	En29.6Wo20.7Fs49.7	En35.7Wo20.1Fs44.2	En28.9Wo20.9Fs50.3	En31.1Wo20.6Fs48.3
χ^2^/ν†	13.8	12.9	12.9	19.6	12.9	15.1

Mineral proportion values in wt.%.

^*^Minerals shown in italics (apatite and Cr-spinel) are based on assumed input values for P_2_O_5_ and Cr_2_O_3_ in similar mare basalts (Papike *et al*.^17^) to more accurately model Ca and Fe. Model mineral component compositions are given in Supplementary Table 7.

^†^χ^2^/ν is a measure of the goodness of fit of the mixing model and is the error-weighted sum of squares of differences between model and actual compositions divided by the number of chemical parameters (oxides) minus the number of mixing components (minerals)^53^.

^‡^Mean_0006 is the average value of CE3-0006_2 and CE3-0006_3. Mean_all is the average value of CE3-0006_2 and CE3-0006_3 and CE3-0008.

## References

[b1] Heiken G. H., Vaniman D. T., French B. M. (eds). Lunar Sourcebook: A User's Guide to the Moon Cambridge University Press (1991).

[b2] Lucey P. G. . in New Views of the Moon Vol. 60, eds Jolliff B. L., Wieczorek M. A., Shearer C. K., Neal C. R. 83–219Mineralogical Society of America (2006).

[b3] Ip W. H., Yan J., Li C. L. & Ouyang Z. Y. Preface: the Chang′e-3 lander and rover mission to the Moon. Res. Astron. Astrophys. 14, 1511 (2014).

[b4] Li C. L. . Analysis of the geomorphology surrounding the Chang′e-3 landing site. Res. Astron. Astrophys. 14, 1514–1529 (2014).

[b5] Xiao L. China's touch on the Moon. Nat. Geosci. 7, 391–392 (2014).

[b6] Xiao L. . A young multilayered terrane of the northern Mare Imbrium revealed by Chang′E-3 mission. Science 347, 1226–1229 (2015). 25766228 10.1126/science.1259866

[b7] Hiesinger H., Jaumann R., Neukum G. & Head J. W. Ages of mare basalts on the lunar nearside. J. Geophys. Res. 105, 29239–29275 (2000).

[b8] Schaber G. G. Lava flows in Mare Imbrium: Geologic evaluation from Apollo orbital photography. Proc. Lunar Planet Sci. Conf. 4, 73–92 (1973).

[b9] Bugiolacchi R. & Guest J. E. Compositional and temporal investigation of exposed lunar basalts in the Mare Imbrium region. Icarus 197, 1–18 (2008).

[b10] Zhao J. . Geologic characteristics of the Chang′E-3 exploration region. Sci. China-Phys. Mech. Astron. 57, 569–576 (2014).

[b11] Hiesinger H., Head J. W., Wolf U., Jaumann R. & Neukum G. Lunar mare basalt flow units: thicknesses determined from crater size-frequency distributions. Geophys. Res. Lett. 29, 89 (2002).

[b12] Peng W. X. . Active particle-induced X-ray Spectrometer for CHANG'E-3 YuTu Rover Mission and its first results. In 45th Lunar and Planetary Science Conference Abstract no. 1699 (2014).

[b13] Fu X. H. . Data processing for the Active Particle-induced X-ray Spectrometer and initial scientific results from Chang′e-3 mission. Res. Astron. Astrophys. 14, 1595–1606 (2014).

[b14] He Z. P. . Operating principles and detection characteristics of the Visible and Near-Infrared Imaging Spectrometer in the Chang′e-3. Res. Astron. Astrophys. 14, 1567–1577 (2014).

[b15] Liu B. . Data processing and preliminary results of the Chang′e-3 VIS/NIR Imaging Spectrometer in-situ analysis. Res. Astron. Astrophys. 14, 1578–1594 (2014).

[b16] Liu B. . Reflectance conversion methods for the VIS/NIR imaging spectrometer aboard the Chang'E-3 lunar rover: based on ground validation experiment data. Res. Astron. Astrophys. 13, 862–874 (2013).

[b17] Papike J. J., Ryder G. & Shearer C. K. in Planetary Materials ed. Papike J. J. 1–234Mineralogical Society of America (1998).

[b18] Burns R. G. Mineralogical Applications of Crystal Field Theory 2nd ed. Cambridge University Press (1993).

[b19] Sunshine J. M. & Pieters C. M. Estimating modal abundances from the spectra of natural and laboratory pyroxene mixtures using the modified Gaussian model. J. Geophys. Res. 98, 9075–9087 (1993).

[b20] Sunshine J. M. & Pieters C. M. Determining the composition of olivine from reflectance spectroscopy. J. Geophys. Res. 103, 13675–13688 (1998).

[b21] Staid M. I. . The mineralogy of late stage lunar volcanism as observed by the Moon Mineralogy Mapper on Chandrayaan-1. J. Geophys. Res. 116, E00G10 (2011).

[b22] Besse S. . Compositional variability of the Marius Hills volcanic complex from the Moon Mineralogy Mapper (M^3^). J. Geophys. Res. 116, E00G13 (2011).

[b23] Varatharajan I., Srivastava N. & Murty S. V. Mineralogy of young lunar mare basalts: assessment of temporal and spatial heterogeneity using M^3^ data from Chandrayaan-1. Icarus 236, 56–71 (2014).

[b24] Clénet H. . A new systematic approach using the Modified Gaussian Model: Insight for the characterization of chemical composition of olivines, pyroxenes and olivine–pyroxene mixtures. Icarus 213, 404–422 (2011).

[b25] Isaacson P. J. & Pieters C. M. Deconvolution of lunar olivine reflectance spectra: Implications for remote compositional assessment. Icarus 210, 8–13 (2010).

[b26] Isaacson P. J. . Remote compositional analysis of lunar olivine-rich lithologies with Moon Mineralogy Mapper (M^3^) spectra. J. Geophys. Res. 116, E00G11 (2011).

[b27] Adams J. B. Visible and near-infrared diffuse reflectance spectra of pyroxenes as applied to remote sensing of solid objects in the solar system. J. Geophys. Res. 79, 4829–4836 (1974).

[b28] Cloutis E. A. & Gaffey M. J. Pyroxene spectroscopy revisited: Spectral-compositional correlations and relationship to geothermometry. J. Geophys. Res. 96, 22809–22826 (1991).

[b29] Klima R. L., Pieters C. M. & Dyar M. D. Spectroscopy of synthetic Mg-Fe pyroxenes I: spin-allowed and spin-forbidden crystal field bands in the visible and near-infrared. Meteorit. Planet. Sci. 42, 235–253 (2007).

[b30] Klima R. L., Dyar M. D. & Pieters C. M. Near-infrared spectra of clinopyroxenes: effects of calcium content and crystal structure. Meteorit. Planet. Sci. 46, 379–395 (2011).

[b31] Sunshine J. M. . High-calcium pyroxene as an indicator of igneous differentiation in asteroids and meteorites. Meteorit. Planet. Sci. 39, 1343–1357 (2004).

[b32] Kanner L. C., Mustard J. F. & Gendrin A. Assessing the limits of the Modified Gaussian Model for remote spectroscopic studies of pyroxenes on Mars. Icarus 187, 442–456 (2007).

[b33] Cloutis E. A., Gaffey M. J., Jackowski T. L. & Reed K. L. Calibrations of phase abundance, composition, and particle size distribution for olivine-orthopyroxene mixtures from reflectance spectra. J. Geophys. Res. 91, 641–11,653 (1986).

[b34] Staid M. I. & Pieters C. M. Mineralogy of the last lunar basalts: results from Clementine. J. Geophys. Res. 106, 27887–27900 (2001).

[b35] Basaltic Volcanism Study Project. Basaltic Volcanism on the Terrestrial Planets Pergamon Press: New York, (1981).

[b36] Neal C. R., Wu Y. Z., Cui X. Z., Peng W. X. & Ping J. S. Regolith at the Chang′e-3 Landing Site: a new type of Mare Basalt Composition. In 46th Lunar and Planetary Science Conference Abstract no. 1641 (2015).

[b37] Prettyman T. H. . Elemental composition of the lunar surface: analysis of gamma ray spectroscopy data from Lunar Prospector. J. Geophys. Res. 111, E12007 (2006).

[b38] Giguere T. A., Taylor G. J., Hawke B. & Lucey P. G. The titanium contents of lunar mare basalts. Meteorit. Planet. Sci. 35, 193–200 (2000).

[b39] Lucey P. G., Blewett D. T. & Jolliff B. L. Lunar iron and titanium abundance algorithms based on final processing of Clementine ultraviolet-visible images. J. Geophys. Res. 105, 20297–20305 (2000).

[b40] Gillis-Davis J. J., Lucey P. G. & Hawke B. R. Testing the relation between UV–VIS color and TiO_2_ content of the lunar maria. Geochim. Cosmochim. Acta 70, 6079–6102 (2006).

[b41] Lucey P. G. Mineral maps of the Moon. Geophys. Res. Lett. 31, L08701 (2004).

[b42] Greenhagen B. T. . Global silicate mineralogy of the Moon from the Diviner Lunar Radiometer. Science 329, 1507–1509 (2010). 20847266 10.1126/science.1192196

[b43] Pieters C. M. . Late high-titanium basalts of the western maria: geology of the Flamsteed region of Oceanus Procellarum. J. Geophys. Res. 85, 3913–3938 (1980).

[b44] Thiessen F., Besse S., Staid M. I. & Hiesinger H. Mapping lunar mare basalt units in mare Imbrium as observed with the Moon Mineralogy Mapper (M^3^). Planet. Space Sci. 104, 244–252 (2014).

[b45] Jolliff B. L., Gillis J. J., Haskin L. A., Korotev R. L. & Wieczorek M. A. Major lunar crustal terranes: surface expressions and crust-mantle origins. J. Geophys. Res. 105, 4197–4216 (2000).

[b46] Haskin L. A., Gillis J. J., Korotev R. L. & Jolliff B. L. The materials of the lunar Procellarum KREEP Terrane: a synthesis of data from geomorphological mapping, remote sensing, and sample analyses. J. Geophys. Res. 105, 20403–20415 (2000).

[b47] Snyder G. A., Taylor L. A. & Neal C. R. A chemical model for generating the sources of mare basalts: combined equilibrium and fractional crystallization of the lunar magmasphere. Geochim. Cosmochim. Acta 56, 3809–3823 (1992).

[b48] Elardo S. M. . The origin of young mare basalts inferred from lunar meteorites Northwest Africa 4734, 032, and LaPaz Icefield 02205. Meteorit. Planet. Sci. 49, 261–291 (2014).

[b49] Wieczorek M. A. & Phillips R. J. The ‘Procellarum KREEP Terrane': Implications for mare volcanism and lunar evolution. J. Geophys. Res. 105, 20417–20430 (2000).

[b50] Hapke B. Theory of Reflectance and Emittance Spectroscopy Cambridge University Press (2005).

[b51] Li S. & Li L. Radiative transfer modeling for quantifying lunar surface minerals, particle size, and submicroscopic metallic Fe. J. Geophys. Res. 116, E09001 (2011).

[b52] Savitzky A. & Golay M. J. Smoothing and differentiation of data by simplified least squares procedures. Anal. Chem. 36, 1627–1639 (1964).

[b53] Korotev R. L., Haskin L. A. & Jolliff B. L. A simulated geochemical rover mission to the Taurus Littrow valley of the Moon. J. Geophys. Res. 100, 14403–14420 (1995).

