# Supplementary material for: Correlated compositional and mineralogical investigations at the Chang′e-3 landing site
Source: Nat Commun. 2015 Dec 22;6:8880. doi: 10.1038/ncomms9880 (PMC4703877; doi:10.1038/ncomms9880)
Supplement: Supplementary Information — Supplementary Figures 1-7, Supplementary Tables 1-8, Supplementary Notes 1-5 and Supplementary References. [file ncomms9880-s1.pdf]

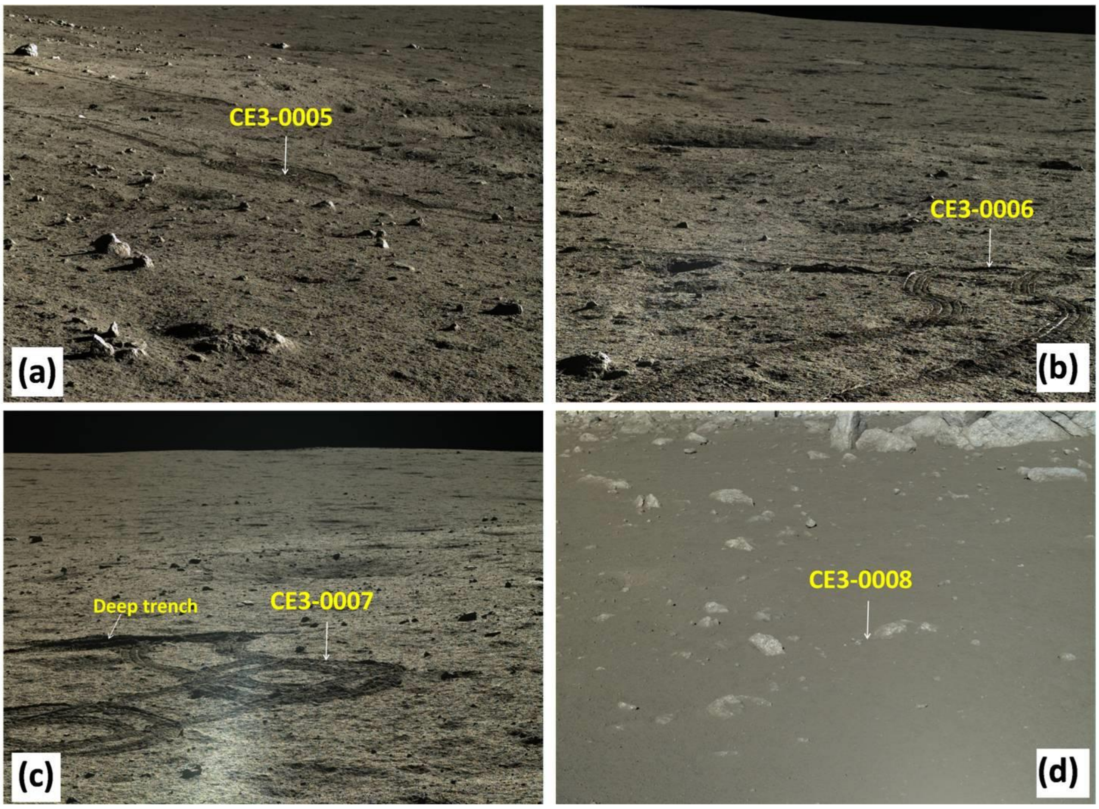

Supplementary Figure 1 Panoramic view of four sites (CE-0005, CE-0006, CE-0007, and CE-0008) measured by APXS and VNIS. Images (a), (b), and (d) were acquired by the Panoramic Camera at the CE3-0007 site, targeting on CE-0005, CE-0006, and CE-0008, respectively. Image (c) was taken by the Panoramic Camera at the CE3-0008 site, focusing on the CE-0007 site. See Supplementary Note 1 for more discussion.

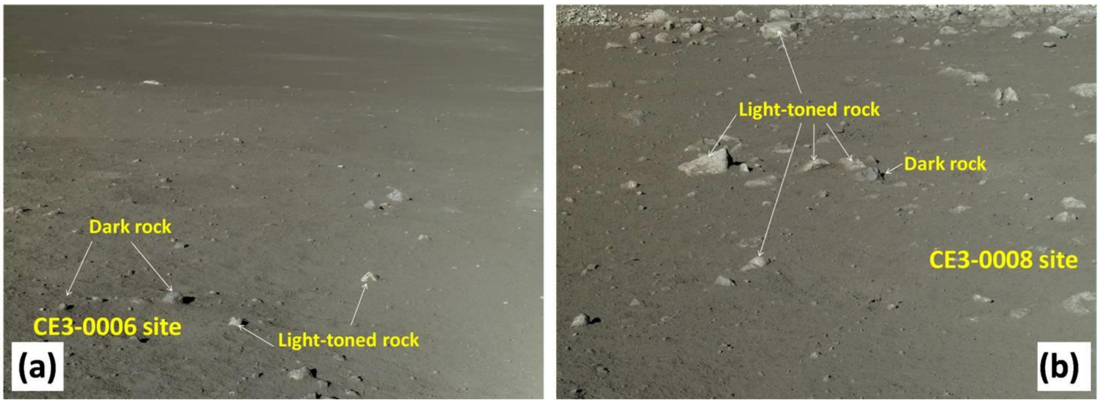

Supplementary Figure 2 Two types of rock, light-toned, coarse-grained rock and dark, fine-grained rock, as seen by the Panoramic Camera at the CE3-0006 site (a) and -0008 site (b). See Supplementary Note 1 for more discussion.

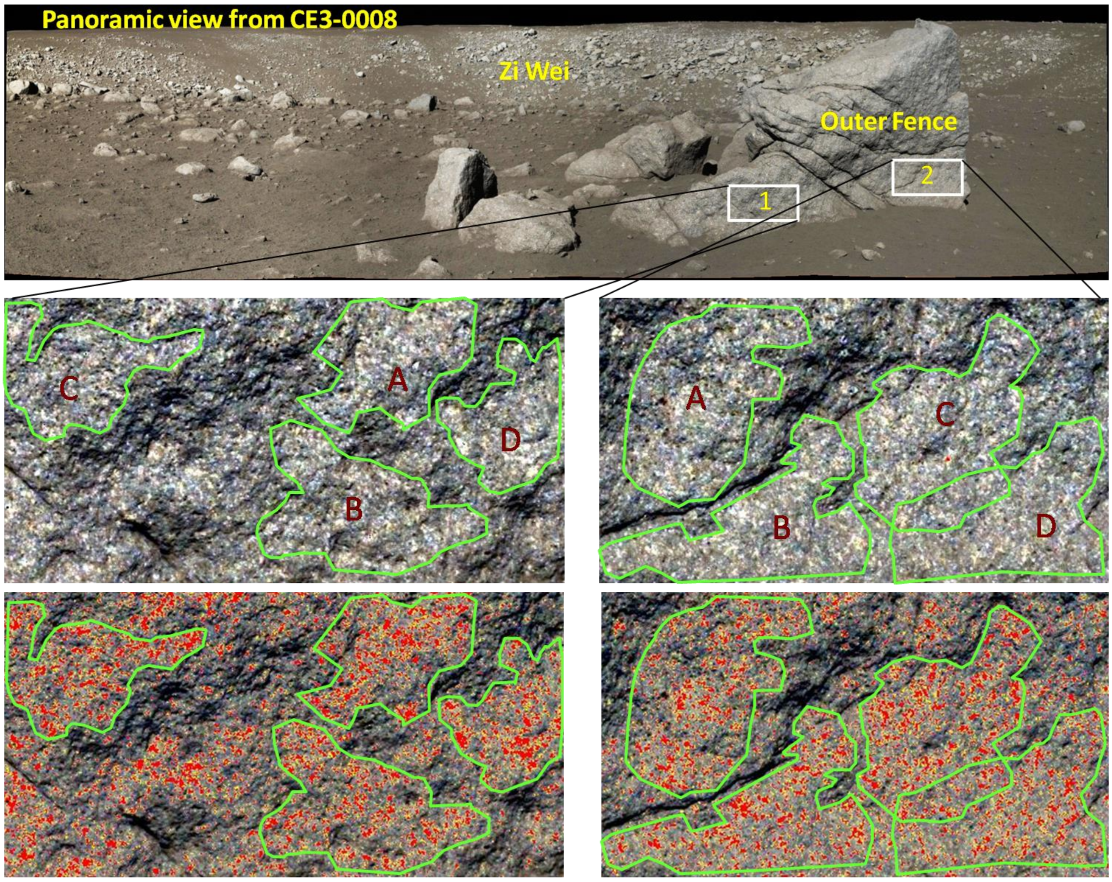

(a)

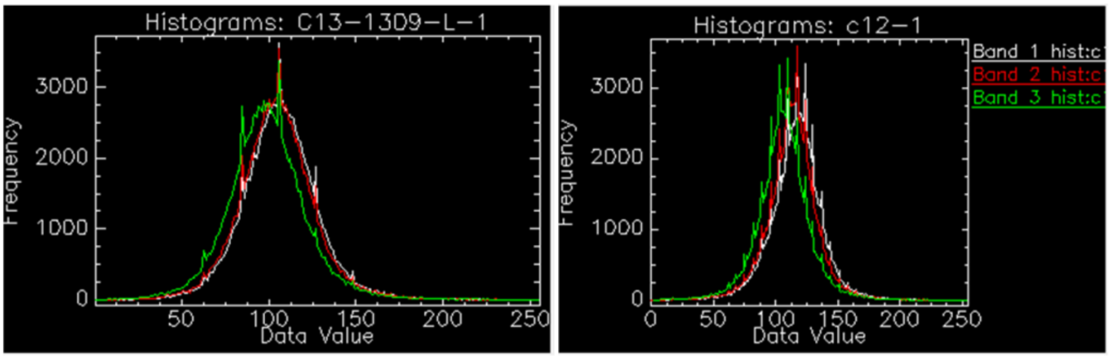

(b)

Supplementary Figure 3 (a) The abundance of coarse-grained plagioclase estimated using the Panoramic Camera image of Outer Fence. We determined the modes of two regions (1 and 2), which contain bright grains, reddish grains, and bluish or greenish grains. Different DN thresholds were set to represent plagioclase grains (Image 1, red: 130, yellow, 125; Image 2, red:125, yellow, 120). (b) Statistics of RGB channels of image 1 and image 2. See Supplementary Note 2 for more discussion.

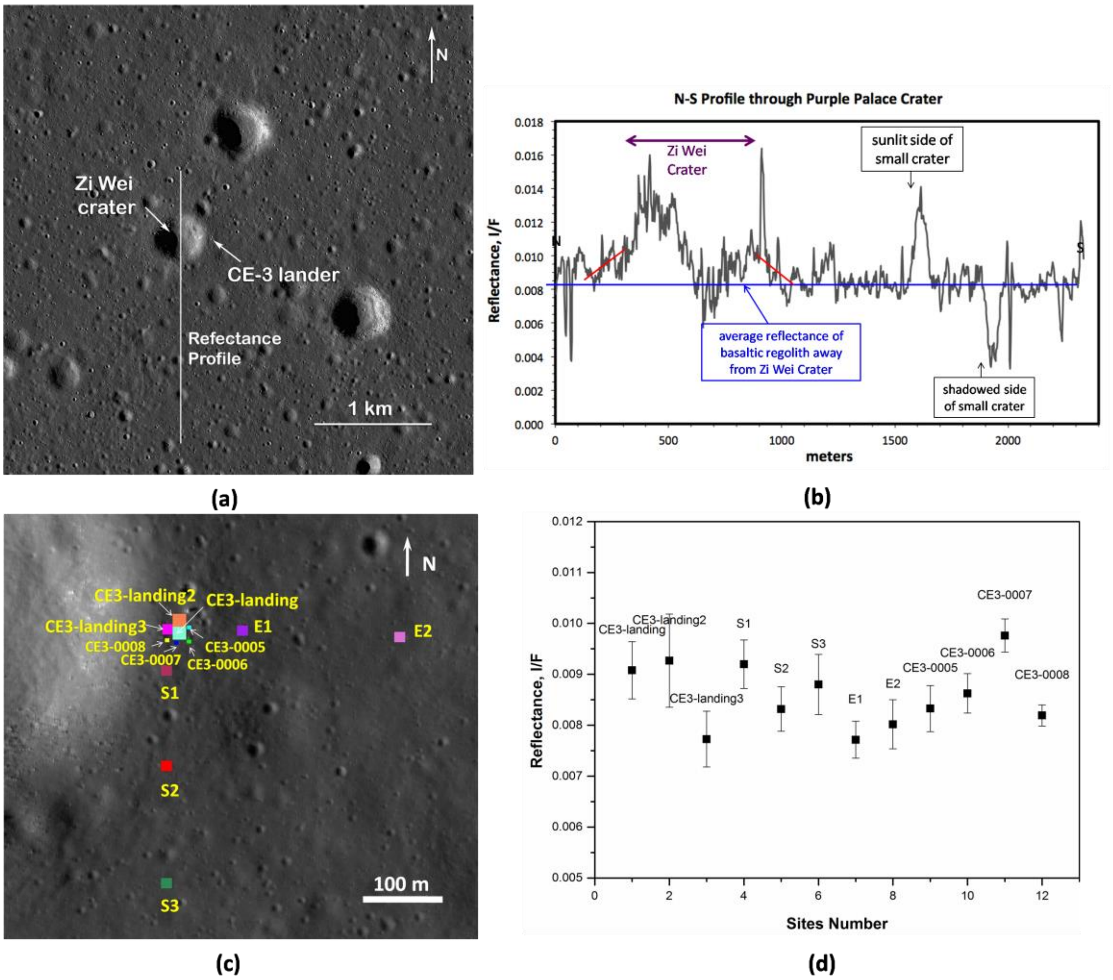

Supplementary Figure 4 Reflectance variations of the lunar regolith near the CE-3 landing site determined by LROC NAC data. See Supplementary Note 3 for more discussion.

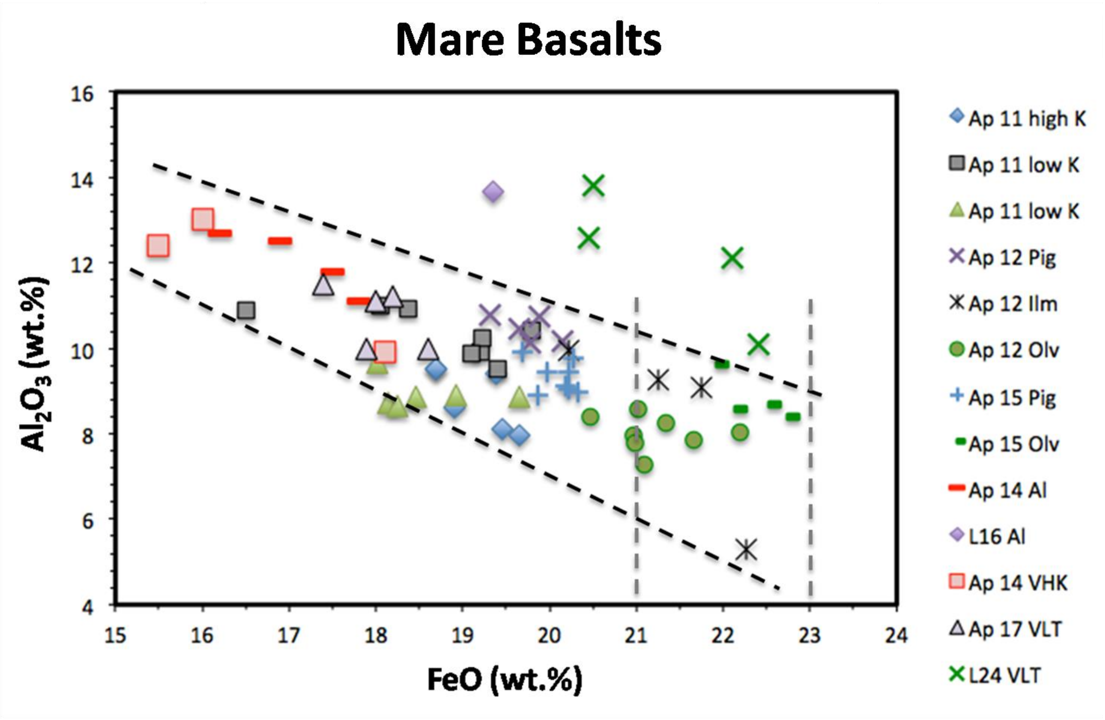

Supplementary Figure 5 Lunar FeO vs. Al_2O_3 in mare basalts from Apollo and Luna basaltic samples (from Papike *et al*., Planetary Materials[1]). See Supplementary Note 4 for discussion.

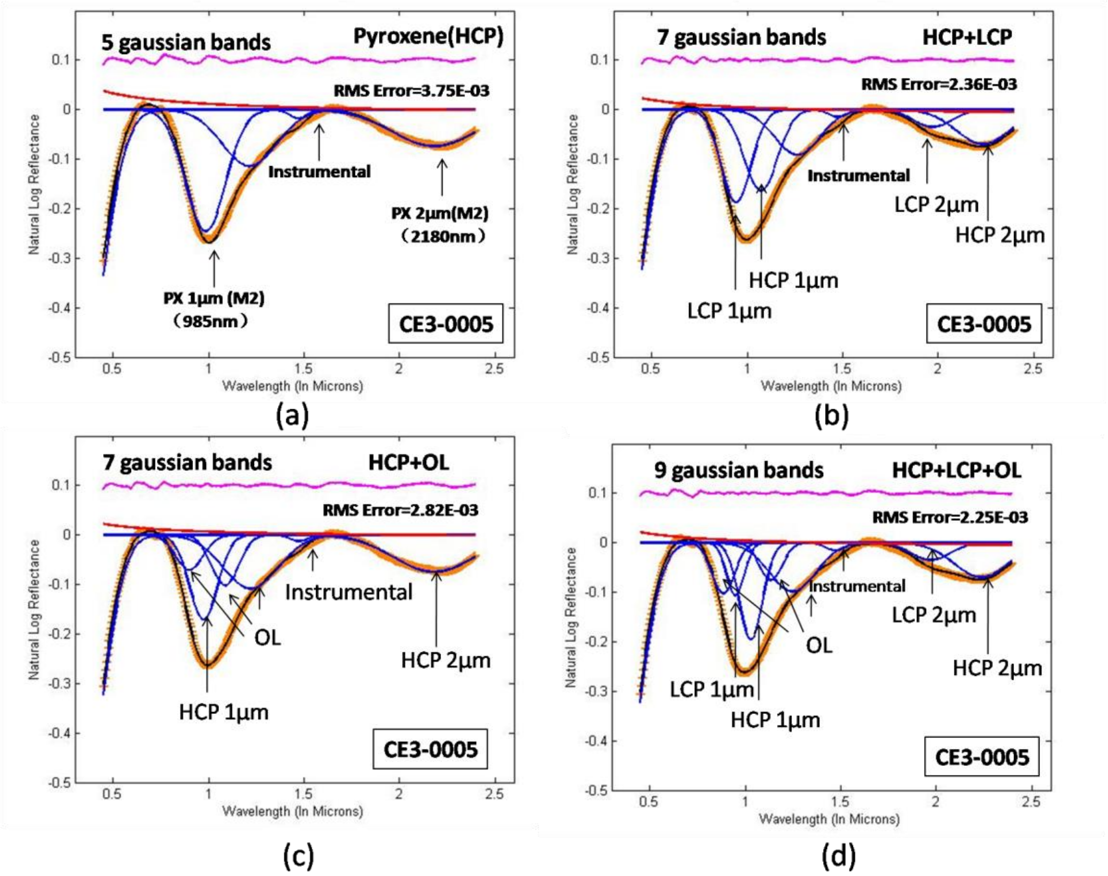

Supplementary Figure 6 Examples of MGM fitting of CE3-0005 spectra. Modelling using pyroxene components: (a), high-Ca pyroxene (HCP) and Low-Ca pyroxene (LCP) mixture (b), HCP and Olivine (OL) mixture (c), and HCP, LCP, OL mixture. See Supplementary Note 5 for details.

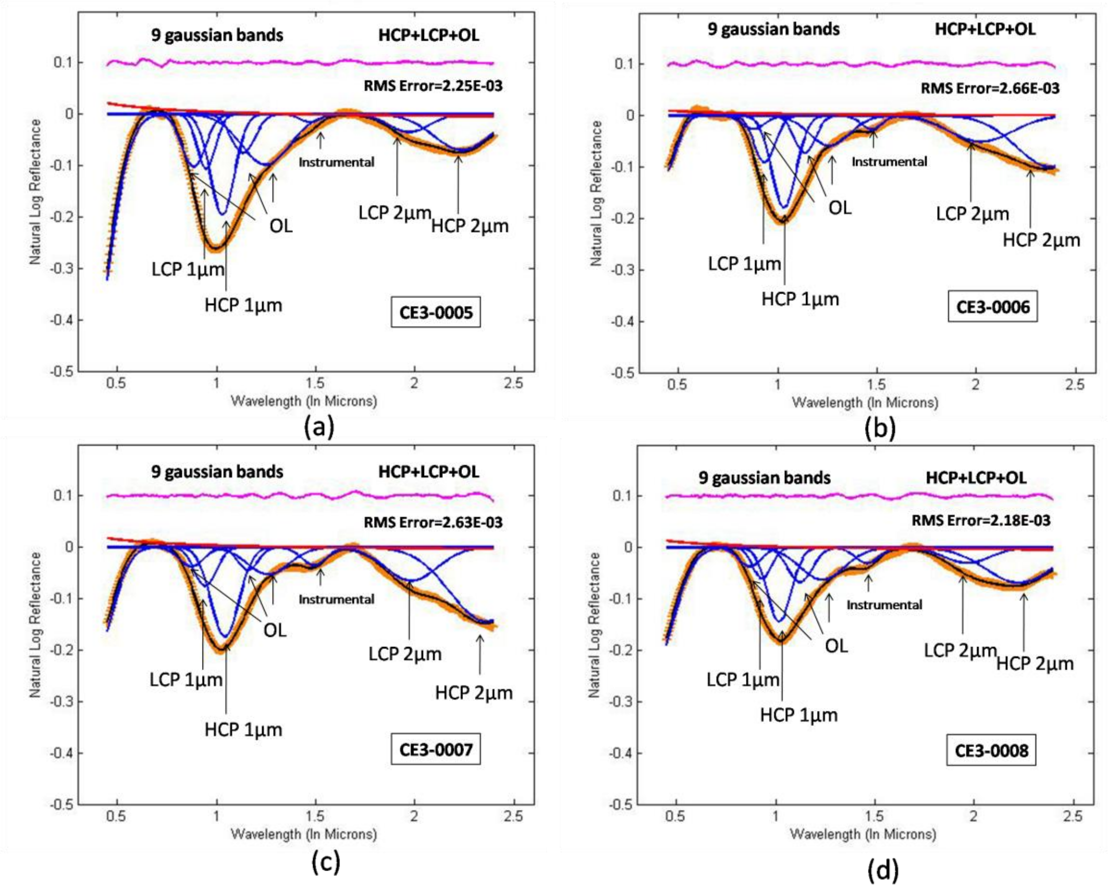

Supplementary Figure 7 Final MGM deconvolution of the VNIS spectra from CE-0005 (a), CE-0006 (b), CE-0007 (c), CE-0005 (d). See Supplementary Note 5 for more explanation.

Supplementary Table 1 Elemental peak-area ratios to Si by CE-3 APXS measurements. Analysis 0006_1 was in the test mode, i.e., with a surface-to-detector separation of ~5 cm. Analysis 0006_2, 0006_3, and 0008 were measured at ~2 cm distance between detector and regolith surface.

Elements/Si (Kα)	Calibration target 1	CE3-0006_1	CE-30006_2	CE3-0006_3	Calibration target 2	CE3-0008
Mg/Si	0.038	0.033	0.026	0.029	0.023	0.030
Al/Si	0.196	0.161	0.155	0.161	0.197	0.159
K/Si	1.145	0.085	0.088	0.083	1.205	0.090
Ca(Kα)/Si	6.049	11.54	11.59	11.81	6.431	11.76
Ca(Kβ)/Si	0.892	1.697	1.698	1.745	0.884	1.710
Ti(Kα)/Si	2.797	7.574	7.684	7.793	3.014	7.929
Ti(Kβ)/Si	0.505	1.201	1.203	1.244	0.537	1.255
Cr/Si	0.005	0.199	0.195	0.199	0.005	0.204
Fe/Si	0.187	0.449	0.464	0.469	0.180	0.484

Supplementary Table 2 CIPW normative mineralogy (in wt.%) of the CE-3 soils measured by APXS.

APXS / CIPW	CE3-0006_1*	CE3-0006_2	CE3-0006_3	CE3-0008	Mean_0006*	Mean_all*
Qtz	0.0	0.0	0.0	0.0	0.0	0.0
Or	0.7	0.7	0.7	0.6	0.7	0.7
*Ab***	*2.5*	*2.5*	*2.5*	*2.5*	*2.5*	*2.5*
An	25.6	24.9	25.5	23.8	25.2	24.8
Di Wo	14.6	15.3	15.1	13.0	15.2	14.5
Di En	5.7	5.1	5.3	5.7	5.2	5.4
Di Fs	9.0	10.8	10.2	7.3	10.5	9.4
Excess Wo	0.0	0.0	0.0	0.0	0.0	0.0
Hy En	6.0	6.6	5.3	1.5	5.9	4.5
Hy Fs	9.4	13.9	10.2	1.9	12.0	8.7
Fo	6.0	2.9	4.7	14.2	3.8	7.3
Fa	10.3	6.8	10.0	19.9	8.4	12.2
Ilm	9.5	9.8	9.8	8.8	9.8	9.5
*Cm***	*0.4*	*0.4*	*0.4*	*0.4*	*0.4*	*0.4*
*Ap***	*0.2*	*0.2*	*0.2*	*0.2*	*0.2*	*0.2*
Sum	100.0	100.0	100.0	100.0	100.0	100.0
Mg/(Mg+Fe)×100	40.0	33.2	35.6	46.0	34.4	38.3

* Analysis 0006_1 was in the test mode. Mean_0006 is the average value of CE3-0006_2 and CE3-0006_3. Mean_all is the average value of CE3-0006_2 and CE3-0006_3 and CE3-0008.

**Values based on assumed typical Na_2O, Cr_2O_3, and P_2O_5 contents.

Supplementary Table 3 Normative mineral contents of the CE-3 soils (in vol.%). Weight percentage norms are converted to volume percentage using average mineral densities.

**ID***	**CE3-0006_1***	**CE3-0006_2**	**CE3-0006_3**	**CE3-0008**	**Mean_0006***	**Mean all***
Sum Plag:	33.5	32.8	33.5	31.8	33.1	32.7
Sum Px:	44.8	51.0	46.1	31.0	48.5	42.7
Sum Ol:	13.8	8.1	12.3	29.7	10.2	16.7
Ilm	6.6	6.8	6.8	6.1	6.8	6.6
Di：	30.9	32.8	32.3	27.9	32.5	31.0
Hy：	13.8	18.2	13.8	3.2	16.0	11.7
Sum En:	11.9	11.9	10.9	7.4	11.4	10.1
Sum Fs:	16.0	21.4	17.8	8.4	19.6	15.9
Sum Wo:	16.8	17.7	17.5	15.2	17.6	16.8
Fo	45.5	38.3	40.8	50.8	39.5	43.3
Fa	54.5	61.7	59.2	49.2	60.5	56.7
Plag An content**	88.4	88.0	88.4	87.7	88.2	88.0

* We combine the diopside (Di) normative components (Di Wo, Di En, Di Fs) and the hypersthene (Hy) normative components (Hy En, Hy Fs), as well as a summed pyroxene component breakdown (sum En, sum Fs, sum Wo). Analysis 0006_1 was in the test mode. Mean_0006 is the average value of CE3-0006_2 and CE3-0006_3. Mean_all is the average value of CE3-0006_2 and CE3-0006_3 and CE3-0008.

** Plag An is defined as An/(An+Ab+Or)×100.

Supplementary Table 4 Relative errors (%) of APXS for different elements derived by ground tests on soils and rock samples.

Elements	Calib_target concentration (wt.%)	Soil relative error*	Rock relative error*
MgO	10.12	0.9	12.7
Al_2O_3	13.47	6.6	13.5
SiO_2	46.2	7.5	3.3
K_2O	1.71	8.7	14.7
CaO	7.15	2.9	5.3
TiO_2	2.05	1.5	5.8
FeO	9.99	9.3	11.2

***** Reports on the Science Validation Experiments of APXS aboard Chang'E-3 in the Chinese Lunar Exploration Program Phase II, by the Departments of Lunar and Deep Space Explorations, National Astronomical Observatories, Chinese Academy of Sciences. CE3-GRAS-CSSY-004-F2, June 2013.

Supplementary Table 5 The area percentage of bright grains in the areas of image 1 and image 2 within Outer Fence. See Supplementary Note 2 for details.

Descriptions	DN Threshold	A(%)	B(%)	C(%)	D(%)	Mean(%)	Standard deviation
**Image 1**	130(red)	13.1	14.7	16.6	14.2	14.7	1.3
	125 (red+yellow)	17.4	21.3	22.6	20.6	20.5	1.9
**Image 2**	125(red)	20.2	15.9	18.2	19.2	18.4	1.6
	120 (red+yellow)	26.7	21.6	24.8	25.3	24.6	1.9

Supplementary Table 6 Starting parameters and Root Mean Square (RMS) errors of MGM analysis. See Supplementary Note 5 for discussion.

**Starting fit**	**CE3-0005**	**CE3-0006**	**CE3-0007**	**CE3-0008**
No. of Bands	9	9	9	9
Band 1 center	403	403	403	403
Band 1 width	328	328	328	328
Band 1 intensity	-0.4	-0.4	-0.48	-0.48
Band 2 center	870	870	870	860
Band 2 width	150	140	150	150
Band 2 intensity	-0.3	-0.3	-0.48	-0.4
Band 3 center	915	915	920	915
Band 3 width	110	120	130	110
Band 3 intensity	-0.2	-0.3	-0.2	-0.1
Band 4 center	970	970	970	970
Band 4 width	140	140	160	140
Band 4 intensity	-0.2	-0.2	-0.3	-0.2
Band 5 center	1050	1050	1050	1030
Band 5 width	120	100	100	130
Band 5 intensity	-0.1	-0.1	-0.1	-0.15
Band 6 center	1250	1250	1250	1230
Band 6 width	250	200	200	230
Band 6 intensity	-0.4	-0.4	-0.4	-0.4
Band 7 center	1400	1400	1450	1400
Band 7 width	100	100	100	100
Band 7 intensity	-0.1	-0.1	-0.1	-0.1
Band 8 center	1900	1900	1900	1900
Band 8 width	200	400	300	300
Band 8 intensity	-0.2	-0.2	-0.1	-0.25
Band 9 center	2270	2270	2270	2270
Band 9 width	300	400	400	400
Band 9 intensity	-0.2	-0.2	-0.1	-0.25
**RMS Error**	2.25E-03	2.66E-03	2.63E-03	2.18E-03

Supplementary Table 7 Input mineral chemistry for mixture modelling of the CE-3 soils (in wt.%). The mineral chemistry was calculated based on the chemical data of endmembers of Apollo mare basalt samples and lunar basaltic meteorites of intermediate TiO_2 contents[1,2,3]. Mineral compositions were selected that would yield appropriate Mg/(Mg+Fe).

(1) CE3_0006_1

	olivine	augite	pigeonite	plagioclase	ilmenite	apatite	Cr-spinel
SiO_2	33.44	48.52	49.07	48.10	0.00	0.00	0.00
TiO_2	0.00	1.69	1.01	0.00	52.38	0.00	6.27
Al_2O_3	0.00	2.55	1.60	32.00	0.28	0.00	11.00
Cr_2O_3	0.13	0.62	0.41	0.00	0.24	0.00	43.80
FeO	48.90	21.07	26.25	1.17	45.16	0.00	33.30
MnO	0.09	0.33	0.41	0.00	0.34	0.00	0.20
MgO	16.96	9.85	11.91	0.08	0.39	0.00	3.94
CaO	0.51	15.34	9.33	17.50	0.00	55.60	0.00
Na_2O	0.00	0.03	0.00	0.40	0.00	0.00	0.00
K_2O	0.00	0.00	0.00	0.20	0.00	0.00	0.00
P_2O_5	0.00	0.00	0.00	0.00	0.00	42.20	0.00
Mg/(Mg+Fe)	0.38	0.45	0.45				

Supplementary Table 7 continued:

(2) CE3_0006_2

	olivine	augite	pigeonite	plagioclase	ilmenite	apatite	Cr-spinel
SiO_2	33.07	48.26	48.43	48.10	0.00	0.00	0.00
TiO_2	0.00	1.55	0.98	0.00	52.38	0.00	6.27
Al_2O_3	0.00	2.26	1.32	32.00	0.28	0.00	11.00
Cr_2O_3	0.12	0.51	0.28	0.00	0.24	0.00	43.80
FeO	51.06	23.44	29.74	1.17	45.16	0.00	33.30
MnO	0.08	0.35	0.44	0.00	0.34	0.00	0.20
MgO	15.20	8.01	9.22	0.08	0.39	0.00	3.94
CaO	0.52	15.58	9.60	17.50	0.00	55.60	0.00
Na_2O	0.00	0.03	0.00	0.40	0.00	0.00	0.00
K_2O	0.00	0.00	0.00	0.20	0.00	0.00	0.00
P_2O_5	0.00	0.00	0.00	0.00	0.00	42.20	0.00
Mg/(Mg+Fe)	0.35	0.38	0.36				

Supplementary Table 7 continued:

(3) CE3-0006_3

	olivine	augite	pigeonite	plagioclase	ilmenite	apatite	Cr-spinel
SiO_2	33.22	48.54	48.55	48.10	0.00	0.00	0.00
TiO_2	0.00	1.70	0.99	0.00	52.38	0.00	6.27
Al_2O_3	0.00	2.57	1.36	32.00	0.28	0.00	11.00
Cr_2O_3	0.12	0.63	0.30	0.00	0.24	0.00	43.80
FeO	50.20	20.90	29.16	1.17	45.16	0.00	33.30
MnO	0.08	0.33	0.43	0.00	0.34	0.00	0.20
MgO	15.90	9.98	9.73	0.08	0.39	0.00	3.94
CaO	0.52	15.32	9.47	17.50	0.00	55.60	0.00
Na_2O	0.00	0.03	0.00	0.40	0.00	0.00	0.00
K_2O	0.00	0.00	0.00	0.20	0.00	0.00	0.00
P_2O_5	0.00	0.00	0.00	0.00	0.00	42.20	0.00
Mg/(Mg+Fe)	0.36	0.46	0.37				

Supplementary Table 7 continued:

(4) CE3_0008

	olivine	augite	pigeonite	plagioclase	ilmenite	apatite	Cr-spinel
SiO_2	34.71	49.00	49.07	48.10	0.00	0.00	0.00
TiO_2	0.00	1.95	1.01	0.00	52.38	0.00	6.27
Al_2O_3	0.00	3.07	1.60	32.00	0.28	0.00	11.00
Cr_2O_3	0.19	0.82	0.41	0.00	0.24	0.00	43.80
FeO	41.57	16.82	26.25	1.17	45.16	0.00	33.30
MnO	0.13	0.28	0.41	0.00	0.34	0.00	0.20
MgO	22.97	13.13	11.91	0.08	0.39	0.00	3.94
CaO	0.44	14.90	9.33	17.50	0.00	55.60	0.00
Na_2O	0.00	0.04	0.00	0.40	0.00	0.00	0.00
K_2O	0.00	0.00	0.00	0.20	0.00	0.00	0.00
P_2O_5	0.00	0.00	0.00	0.00	0.00	42.20	0.00
Mg/(Mg+Fe)	0.50	0.58	0.45				

Supplementary Table 8 Identification information for the images and spectral data used in the figures. The Chang'e data (including Chang'e-1, Chang'e-2, and Chang'e-3) presented in this paper are hosted at http://moon.bao.ac.cn. The LROC NAC image was downloaded from http://lroc.sese.asu.edu/data/.

Figure 1 (a)	Global CCD image of Chang'e-1
Figure 1 (b)	Global CCD image of Chang'e-2
Figure 1 (c)	LROC NAC image (LROC NAC M1142582775R).
Figure 1 (d)	CE3_BMYK_LCAM-2960_SCI_N_20131214131003_20131214131003_0001_A.2A
Figure 1 (e) & Supplementary Figure 3	CE3_BMYK_PCAML-C-012_SCI_N_20140113191206_20140113191206_0008_A.2C
	CE3_BMYK_PCAML-C-013_SCI_N_20140113191309_20140113191309_0008_A.2C
	CE3_BMYK_PCAML-C-014_SCI_N_20140113191411_20140113191411_0008_A.2C
	CE3_BMYK_PCAML-C-015_SCI_N_20140113191514_20140113191514_0008_A.2C
Figure 2 (a)	CE3_BMYK_PIXS-E_SCI_N_20131221124501_20131223174500_0005_A.2B
	CE3_BMYK_PIXS-E_SCI_N_20131223174501_20131226000000_0006_A.2B
Figure 2 (b-d)	Compositions of soils from Apollo and Luna missions (Lucey *et al*., 2006)[2]
Figure 3 & Supplementary Figure 6 & 7	CE3_BMYK_VNIS-CD_SCI_N_20131223021010_20131223021010_0005_A.2B
	CE3_BMYK_VNIS-CD_SCI_N_20131224013542_20131224013542_0006_A.2B
	CE3_BMYK_VNIS-CD_SCI_N_20140112144940_20140112144940_0007_A.2B
	CE3_BMYK_VNIS-CD_SCI_N_20140114163112_20140114163112_0008_A.2B
	CE3_BMYK_VNIS-SD_SCI_N_20131223021320_20131223021320_0005_A.2B
	CE3_BMYK_VNIS-SD_SCI_N_20131224013852_20131224013852_0006_A.2B
	CE3_BMYK_VNIS-SD_SCI_N_20140112145252_20140112145252_0007_A.2B
	CE3_BMYK_VNIS-SD_SCI_N_20140114163422_20140114163422_0008_A.2B
Supplementary Figure 1	CE3_BMYK_PCAMR-C-007_SCI_N_20140112131750_20140112131750_0007_A.2C
	CE3_BMYK_PCAMR-C-024_SCI_N_20140112133531_20140112133531_0007_A.2C
	CE3_BMYK_PCAMR-C-026_SCI_N_20140112133736_20140112133736_0007_A.2C
	CE3_BMYK_PCAMR-C-027_SCI_N_20140113192727_20140113192727_0008_A.2C
Supplementary Figure 2	CE3_BMYK_PCAMR-C-008_SCI_N_20140112131852_20140112131852_0007_A.2C
	CE3_BMYK_PCAMR-C-023_SCI_N_20131224191640_20131224191640_0006_A.2C
Supplementary Figure 4	LROC NAC image (LROC NAC M1142582775R).
Supplementary Figure 5	Lunar Samples (Ch. 5), in Planetary Materials (Papike *et al*., 1998) [1]

Supplementary Note 1:

Description of the four sites and nearby rock types

Supplementary Figure 1 shows the Panoramic view of four compositional sites. In general, CE-0005 and CE-0008 are more blocky with more rocks than the other two sites. Especially at the CE-0007 site, rover tracks and the deep trench made by the rover suggests a thicker regolith than at the other sites.

Two types of rocks were observed by Yutu during its traverse: one is the dominant light-toned, coarse-grained basalt (similar to Outer Fence, which is ~4 m long by 1.5 m high), and the other is darker, fine-grained basalt. The CE3-0006 regolith surface appears to be smoother than the CE3-0008 site, which is expected, owing to the proximity of CE3-0008 to the Zi Wei crater. The CE3-0008 regolith has more coarse rock fragments from crater ejecta. The chemical composition may vary but the color may be due to the texture (grain size) of the rock. Both factors may account for the observations, but we prefer the latter on the basis of the false-color images and Panoramic Camera data. The upper parts of the lava flow should be parts of the flow that underwent rapid cooling, leading to a fine-grained rock texture, whereas the coarse-grained rock would come from a deeper level of the flow and from deeper in Zi Wei crater.

Given the limitation of only two soil samples being measured by APXS (CE3-0006 and -0008), if we assume that the soils derive only from the local basalts and that these basalts are all from the same (upper-most, Eratosthenian) flow unit, then perhaps the analyses reflect a simple fractional crystallization trend of slightly more magnesian and olivine-rich (corresponding to the light-toned coarse-grained rock, Supplementary Figure 2) to more iron and pyroxene-rich lava (corresponding to the CE3-0006 soil and related to the dark, fine-grained rock, Supplementary Figure 2).

Supplementary Note 2:

Estimation of plagioclase abundance by photo-image analysis

We estimated the abundance of plagioclase in one of the nearby boulders, Outer Fence (Supplementary Figure 3), at the landing site, which was likely excavated from the ~450 m diameter Zi Wei crater west of the landing site. This boulder is ~1.5 m tall and dust appears to be minor or absent, thus it is conducive to image analysis. As shown in Supplementary Figure 3, the bright grains likely represent plagioclase with high albedo. The reddish, bluish, or greenish-colored grains are most likely the mafic minerals of these rocks. We determined the pixels' DN values and the threshold of each image. The statistics of the RGB channels are shown in Supplementary Figure 3. The data show a single-mode distribution of image DN values, suggesting the DN variation of the rock is not abrupt. However, we still find a step of ~20 DN for the bright grains compared with surrounding pixels. For Image 1, the threshold of the blue channel DN is 130, indicated by the red pixels in the lower image. For Image 2, the threshold of the blue channel DN is 125. The red pixels of both images make up ~17% (14.7±1.3% and 18.4±1.6%, respectively) of the images. This percentage provides a lower limit estimate of volume percentage of plagioclase in the rock.

In order to accommodate the micro-topography of the rock surface, we subdivided the two images into A-D areas respectively, and we also lowered the thresholds to 125 for Image 1 and 120 for Image 2. The added pixels are shown as yellow in Supplementary Figure 3 and the results are shown in Supplementary Table 5. The percentages of A, B, C, and D areas in each image show relatively small variations, implying the volume of phenocryst plagioclase in the rock does not vary significantly. Our statistics indicate that the range of phenocryst plagioclase may be from 14.7±1.3% to 24.6±1.9% in volume if we assume two thresholds of DNs. The uncertainty of this photo-image statistical analysis based on variability is estimated to be ±4% (one standard deviation of all the four selected areas with two thresholds (Supplementary Table 5)). However, we cannot estimate the fine grains of plagioclase in the rock by this method. Note that the values of DN are also influenced by the roughness of the rock and the observation/illumination geometry, thus one cannot fully capture the mineral mode of plagioclase in this way. In any case, this image-based analysis provides a constraint on the mineral mode of plagioclase. Our APXS data-reduction results suggest ~33 vol.% of plagioclase in the soils of the landing site. This value is in general agreement with the photo-based results here (~20±4 vol.% on average, as a lower limit), considering that the undetermined abundance of fine-grained plagioclase must be added to this value.

In summary, our photo-image analysis suggests that plagioclase phenocrysts make up ~20 vol.% of the rock. If the rocks are richer in Al_2O_3 than indicated by our APXS data reduction (e.g., ~12.6 wt.%[4]), then the plagioclase content would be over ~40 vol.%.

Supplementary Note 3:

Albedo variations of the lunar soils near CE-3 landing site

We examined reflectance variations of the lunar regolith near the CE-3 landing site by using LROC NAC image data. We compared the average reflectance of the regional basaltic regolith with reflectance values for different areas at the landing site that are relatively flat and thus comparable.

Supplementary Figure 4 a and b shows variations in reflectance of soils near the CE-3 landing site. The image shown in (a) is centred at 44.1223ºN and 340.5000ºE, and extracted from NAC image M1142582775R, map in sinusoidal projection (incidence = 77º, emission = 2º, phase = 75º). Most of the variance in reflectance is associated with impact craters, but there is also an increase of reflectance on approach to the rim of Zi Wei crater. The mature surrounding basaltic regolith surface has an average I/F value of 0.0082 whereas the rim of Zi Wei crater rises to ~0.010. We selected 11 sites near the CE-3 landing and traverse sites, as shown in Supplementary Figure 4 c. Typical 8×8 pixel homogeneous areas were selected to average the reflectance values in order to eliminate small-scale topographic effects, except for the two CE-3-landing sites (CE-3-landing and CE-3-landing2, 10×10) and the four traverse sites (3×3). The values more-or-less occur within the range of 0.008 to 0.010.

Those sites near the Zi Wei crater rim (CE-3 landing, E1, S1, as shown in Supplementary Figure 4 c,d) are very similar to the soils ~200 m farther away (S3 and E2). The darkening effect of space weathering on the surrounding regolith away from Zi Wei crater is subtle in the NAC image, indicating that the ejecta, from which the soils derived (e.g., CE-3-landing, S1) near the crater rim is compositionally similar to the regolith derived on top of the Eratosthenian lava flow away from the crater (e.g., S3, E2). If the soils at the CE-3 site had derived from a deeper, more aluminous basalt[4], there would likely be a more obvious increase in reflectance between the ejecta and surrounding regolith.

Supplementary Note 4:

Al_2O_3 content of CE-3 landing site based on experience of lunar samples

The total amount of plagioclase one would expect from a plagioclase-saturated residual melt is around 27-30 wt.%, based on norms of a number of Apollo 15 pigeonite and Apollo 12 ilmenite basalts as listed in Planetary Materials[1]. That range is equivalent to about 33-35 vol.%. As indicated by FeO vs. Al_2O_3 in mare basalts (Supplementary Figure 5), for the high-Fe basalts in the range 21-23 wt.%, the most likely range of Al_2O_3 (indicated by dashed lines) is 7-10 wt.%. We exclude the Luna 24 VLT basalts because they have very low Ti contents and rather exceptional compositions.

If the CE-3 basalts follow trends defined by the Apollo basalts, we would expect that for the high FeO indicated by the CE-3 APXS, Al_2O_3 in the range of 7-10 wt.% is most likely.

Supplementary Note 5:

MGM analysis

We applied the Modified Gaussian Model (MGM)[5,6] to deconvolve the spectral bands as measured by the CE-3 VNIS. We have attempted to use different mineral assemblages(e.g., HCP, HCP+LCP, HCP+OL, HCP+LCP+OL) and various numbers of Gaussian bands (e.g., 4, 5, 6, 7, 8, and 9, etc.) to deconvolve the overlapping absorption features of the CE-3 VNIS spectra. Figure S6 shows examples of different numbers of Gaussian bands used in our MGM modelling. Note that an instrumental artefact peak (~1450 nm) is required for better spectral deconvolution.

One of our major considerations is the mineral components leading to the spectral parameter variations of individual absorption bands (e.g., 1 μm and 2 μm band in VNIS). Generally, for the 1 μm and 2 μm features for a single pyroxene component, their derived spectral band-area ratio should be similar ($A_{1μm}$/ $A_{2μm} \approx 1.0$). From Supplementary Figure 6a we find that if using only pyroxene components, the 1 μm and 2 μm features have very different area ratios ($A_{1μm}/A_{2μm}$=1.9 for Supplementary Figure 6a), thus it is not reasonable to deconvolve the spectra using only pyroxene. However, the spectral fitting result of Supplementary Figure 6a suggests the major component of the CE3-0005 soil is high-Ca pyroxene (HCP, Wo>30) (1 μm feature at 985 nm and 2 μm feature at 2180 nm). For Supplementary Figure 6b, the band area ratio of HCP and LCP (Wo<30) are too high ($A_{1μm}/A_{2μm}$=4.2 for LCP and $A_{1μm}/A_{2μm}$=1.6 for HCP). Although the MGM fits of Supplementary Figure 6b are mathematically good fits, the results shown in Supplementary Figure 6b are not meaningful in terms of mineralogy because variations in proportions of HCP and LCP pyroxenes in one spectrum are opposite, i.e., the strength of LCP 1 μm features are stronger than those of HCP, whereas the 2 μm bands of LCP are less than those of HCP. Thus it is unreasonable to use only HCP and LCP components in the MGM analysis. Considering the soils should be rich in HCP (based on the first spectroscopic check of VNIS spectra as discussed above for Supplementary Figure 6a as well as APXS data), we find that another possibility is a combination of HCP and OL as indicated in Supplementary Figure 6c; however, the derived band centres (899 nm, 1086 nm, i.e., Fe-rich olivine) of Supplementary Figure 6c also contradict the location of the 1221 nm band that suggests an Mg-rich olivine.

Thus we are compelled to use the most complex HCP+LCP+OL mixture to perform MGM modelling. The best result of MGM deconvolution of the CE-0005 soil is shown in Supplementary Figure 6d, which obeys the rules stated above for checking the spectroscopic assignments (realistic combinations) of each mineral component, and they show consistent results with APXS as well (as discussed in the main text).

The starting parameters of MGM are shown in Supplementary Table 6. The final MGM results are shown in Supplementary Figure 7. The software was downloaded from RELAB (http://www.planetary.brown.edu/mgm/).

The precise location of the M2 band (~1050 nm) of olivine in the VNIS spectrum is difficult to determine via MGM deconvolution. Three factors can be influential: (1) overlap with the 1 μm (M1) band (~1030 nm) of HCP; (2) Fe-rich olivine would have an M2 band with low intensity and a shifted central position (Sunshine and Pieters, 1998)[6] to 1080 nm that can cause overlap with the M2 band shoulder (~1150 nm) of Fe-rich pyroxene; and (3) pyroxene M1 bands would dominate the 1 μm band from a mineral mixture whose olivine content is less than 50 vol.%[7].

Note that there are substantial uncertainties[8] (e.g., band positions and widths) in MGM modelling of VNIS spectra if different parameters (e.g., wavelength range, No. of bands, and/or band parameters as shown in Supplementary Table 6) are chosen. In order to find the best MGM modelling results with the lowest uncertainties, we tested many different mineral mixtures and spectral parameters (different numbers of bands, band positions, band widths, and band strengths), guided by chemical and mineralogical constraints from APXS results. The results shown in Supplementary Figure 7 have the lowest uncertainties among all attempted combinations, and at the same time, the best matches with standard spectra of mafic mineral endmembers were achieved.

The MGM analysis of pyroxene mixtures with laboratory and OMEGA data determined that the relative pyroxene abundance (LCP/(HCP+LCP)) can be constrained to ±10% (Kanner *et al*., 2007)[8]. For MGM analysis of laboratory olivine, compositional estimation can be accurate to within ~5–10% (5–10 Fo units) (Sunshine and Pieters, 1998; Isaacson and Pieters, 2010)[6,9]. When the remotely-sensed M^3 data with lower signal-to-noise is applied, the MGM composite error would be on the order of 20 Fo units (Isaacson *et al*., 2011)[10]. As mentioned above, the uncertainties of our MGM analysis of CE-3 VNIS data have been constrained based on the correlated chemical data from the CE-3 APXS. Therefore, the composite errors of MGM analysis in this study, although difficult to quantify, would be the same range or even smaller if considering the higher quality data obtained in-situ by CE-3 VNIS compared to remotely acquired data with increased noise level and decreased spectral resolution by lunar orbital spectrometers.

Supplementary References

1. Papike, J. J., Ryder, G. & Shearer, C. K. Lunar Samples (Ch. 5), in *Planetary Materials*, edited by J. J. Papike, pp. 1-234, Mineralogical Society of America, Washington, D.C., USA. (1998)
2. Lucey, P. G., *et al*. in *New Views of the Moon* Vol.60 (eds Jolliff, B. L., Wieczorek, M. A., Shearer, C. K., & Neal, C. R.) 83-219 (Mineralogical Society of America, 2006).
3. Elardo, S. M., *et al*. The origin of young mare basalts inferred from lunar meteorites Northwest Africa 4734, 032, and LaPaz Icefield 02205. *Meteorit. Planet. Sci.*, **49**, 261-291 (2014).
4. Neal, C. R., Wu, Y. Z., Cui, X. Z., Peng, W. X., & Ping, J. S. Regolith at the Chang'e-3 Landing Site: A New Type of Mare Basalt Composition. *Lunar Planet Sci. 46*, Abstract No. 1641 (2015).
5. Sunshine, J. M., & Pieters, C. M. Estimating modal abundances from the spectra of natural and laboratory pyroxene mixtures using the modified Gaussian model. *J. Geophys. Res.*, **98,** 9075-9087 (1993).
6. Sunshine, J. M., & Pieters, C. M. Determining the composition of olivine from reflectance spectroscopy. *J. Geophys. Res.*, **103**, 13,675-13,688 (1998).
7. Cloutis, E. A., Gaffey, M. J., Jackowski, T. L., & Reed, K. L. Calibrations of phase abundance, composition, and particle size distribution for olivine-orthopyroxene mixtures from reflectance spectra. *J. Geophys. Res.***, 91**, 11641-11653 (1986).
8. Kanner, L. C., Mustard, J. F. & Gendrin, A. Assessing the limits of the Modified Gaussian Model for remote spectroscopic studies of pyroxenes on Mars. *Icarus*, **187**, 442-456 (2007).
9. Isaacson, P. J., & Pieters, C. M. Deconvolution of lunar olivine reflectance spectra: Implications for remote compositional assessment. Icarus, **210**, 8-13 (2010).
10. Isaacson, P. J., *et al*. Remote compositional analysis of lunar olivine-rich lithologies with Moon Mineralogy Mapper (M^3) spectra. *J. Geophys. Res.*, **116**, E00G11 (2011).
